# Duplicate divergence of two bacterial small heat shock proteins reduces the demand for Hsp70 in refolding of substrates

**DOI:** 10.1371/journal.pgen.1008479

**Published:** 2019-10-25

**Authors:** Igor Obuchowski, Artur Piróg, Milena Stolarska, Bartłomiej Tomiczek, Krzysztof Liberek

**Affiliations:** Department of Molecular and Cellular Biology, Intercollegiate Faculty of Biotechnology UG-MUG, University of Gdansk, Gdansk, Poland; The University of Texas Health Science Center at Houston, UNITED STATES

## Abstract

Small heat shock proteins (sHsps) are a conserved class of ATP-independent chaperones that bind to aggregation-prone polypeptides at stress conditions. sHsps encage these polypeptides in assemblies, shielding them from further aggregation. To facilitate their subsequent solubilization and refolding by Hsp70 (DnaK) and Hsp100 (ClpB) chaperones, first, sHsps need to dissociate from the assemblies. In most γ-proteobacteria, these functions are fulfilled by a single sHsp (IbpA), but in a subset of *Enterobacterales*, a two-protein sHsp (IbpA and IbpB) system has evolved. To gain insight into the emergence of complexity within this chaperone system, we reconstructed the phylogeny of γ-proteobacteria and their sHsps. We selected proteins representative of systems comprising either one or two sHsps and analysed their ability to form sHsps-substrate assemblies. All the tested IbpA proteins, but not IbpBs, stably interact with an aggregating substrate. Moreover, in *Escherichia coli* cells, *ibpA* but not *ibpB* suppress the growth defect associated with low DnaK level, which points to the major protective role of IbpA during the breakdown of protein quality control. We also examined how sHsps affect the association of Hsp70 with the assemblies at the initial phase of disaggregation and how they affect protein recovery after stress. Our results suggest that a single gene duplication event has given rise to the sHsp system consisting of a strong canonical binder, IbpA, and its non-canonical paralog IbpB that enhances sHsps dissociation from the assemblies. The cooperation between the sHsps reduces the demand for Hsp70 needed to outcompete them from the assemblies by promoting sHsps dissociation without compromising assembly formation at heat shock. This potentially increases the robustness and elasticity of sHsps protection against irreversible aggregation.

## Author summary

Small heat shock proteins (sHsps) are a class of molecular chaperones playing an important role in maintaining cell proteostasis. Their most widespread and evolutionarily conserved function is binding to denaturing polypeptides. Small Hsps shield their substrates from further aggregation until conditions are favourable for their refolding by chaperones from the Hsp70 and Hsp100 families. To exert this function, at stress conditions, oligomeric sHsps dissociate into dimers and scavenge partially unfolded substrates, forming assemblies containing both substrate proteins and sHsps. Substrate proteins in such assemblies are refolding-competent. Later, when a cell recovers from stress, sHsps need to dissociate from the assemblies to make the substrates available for the disaggregating and refolding chaperones. Most bacteria possess one sHsp-encoding gene. However, their single sHsp is burdened with a trade-off: on one hand, it has to rapidly associate with the misfolding proteins, on the other, it needs to dissociate from them to allow effective disaggregation. With phylogenetic and biochemical approaches, we analysed a two-sHsp system distinctive of the *Enterobacterales* order, unravelling a potential evolutionary advantage granted by functional cooperation between the two sHsps. Our results indicate that after a gene duplication event, one sHsp specialized in tight substrate binding, whereas another sHsp became important for efficient dissociation of both sHsps to enable recovery of proteins trapped in the assemblies.

## Introduction

When a cell is exposed to heat shock, intracellular proteins aggregate, which results in the imbalance of protein homeostasis (proteostasis) [1, 2]. The presence of the protein quality control network restores the proteostasis by counteracting misfolding and by processing aggregated polypeptides [1, 3]. These polypeptides are either degraded by cellular proteases or disaggregated by a chaperone system consisting of three major components. This system includes small heat shock proteins (sHsps), the Hsp70 chaperone system (DnaK with DnaJ and GrpE cochaperones) and an Hsp100 disaggregase (ClpB) [4, 5]. Upon stress, small Hsps become highly abundant in a cell, where they drive protein aggregation toward reactivation-prone assemblies [6–9]. The assemblies are recognized by Hsp70 [10, 11] and further processed by both Hsp70 and Hsp100, which leads to extraction of single polypeptides from aggregates and their subsequent refolding [11, 12].

Being present in all kingdoms of life, small Hsps constitute the first line of proteostasis defence. Members of this protein family are characterized by low molecular mass (12–43 kDa) and share universal structural organization consisting of a ~100 aa α-crystallin domain (conserved β-sandwich fold) flanked by unstructured N- and C-termini of variable length [13]. sHsps form dynamic oligomeric species (up to 32 subunits), which dissociate into smaller forms in a temperature-dependent manner [14–16]. This phenomenon is widely recognized as a mechanism that regulates their activity, namely binding to partially unfolded client proteins at heat shock conditions [17–20]. In Bacteria, Archaea and unicellular Eukaryota, there are usually one or two sHsp-coding genes, whereas in complex, multicellular organisms this number is much higher: 10 in human and even 19 in *Arabidopsis thaliana* [13]. In bacteria sHsps are generally involved in the response to heat shock, therefore deletion of sHsps-coding genes results in the temperature-sensitive phenotype [21, 22] or decreases cell viability during prolonged growth under high temperatures [20].

Upon heat stress, deoligomerized–activated sHsps scavenge misfolded client proteins, encaging them in assemblies containing both client proteins and sHsps. Such assemblies are smaller than amorphous aggregates and store client proteins in their near-native conformations, shielding them from further aggregation [7, 9, 23]. sHsps-clients assemblies are more potent disaggregation substrates for Hsp70-Hsp100 than amorphous aggregates formed in the absence of sHsps [9, 24]. To start disaggregation, sHsps need to be outcompeted from the assemblies' surface by Hsp70 [9]. Next, assembly-associated Hsp70 recruits the Hsp100 disaggregase to allow efficient extraction and refolding of client proteins [25–28].

There are two sHsp family members in *Escherichia coli*: IbpA and IbpB. They were originally identified as inclusion body-associated proteins [29] and later found to be associated with heat shock-induced protein aggregates [30]. Both proteins share the structural and oligomeric features characteristic for sHsps [31–33]. Strains lacking IbpA and IbpB are more sensitive to high temperature and accumulate significantly higher levels of protein aggregates after the exposure to long-term severe heat shock [20]. IbpA and IbpB share 63% amino acid sequence similarity (48% identity) but are functionally diverse. *In vitro*, heat-denatured luciferase in the presence of IbpA and IbpB produces characteristic IbpAB-luciferase assemblies, which show substantial increase in the Hsp70-Hsp100-mediated refolding efficiency compared to luciferase aggregates alone. When analysed separately, IbpA, in contrast to IbpB, is able to form assemblies with aggregating luciferase [23]. Surprisingly, *in vitro* disaggregation and refolding of luciferase from IbpA-luciferase assemblies was less efficient than from luciferase aggregates [23, 24].

In this study, we focused on specific functional interplay between two sHsps, IbpA and IbpB, by analysing their ability of directing protein aggregation towards assemblies formation and in consequence improved substrates’ refolding potential. Our results suggest that IbpA underwent a gene duplication event at the base of *Enterobacterales*, and both resulting genes became functionally divergent. IbpA specialized in efficient substrate binding upon aggregation (holdase activity) and the second post-duplication sHsp (IbpB) became crucial for sHsps release from the assemblies prior to disaggregation step. In other bacteria possessing only one sHsp gene these functions are fulfilled by a single IbpA-like protein. We show that the two-sHsp chaperone system, in contrast to the single sHsp, allows for substantially easier release of sHsps from assemblies without compromising their formation and ensures lower demand for the Hsp70 chaperones in disaggregation and refolding.

## Results

### Evolutionary history of sHsps in γ-proteobacteria

**Fig 1:**
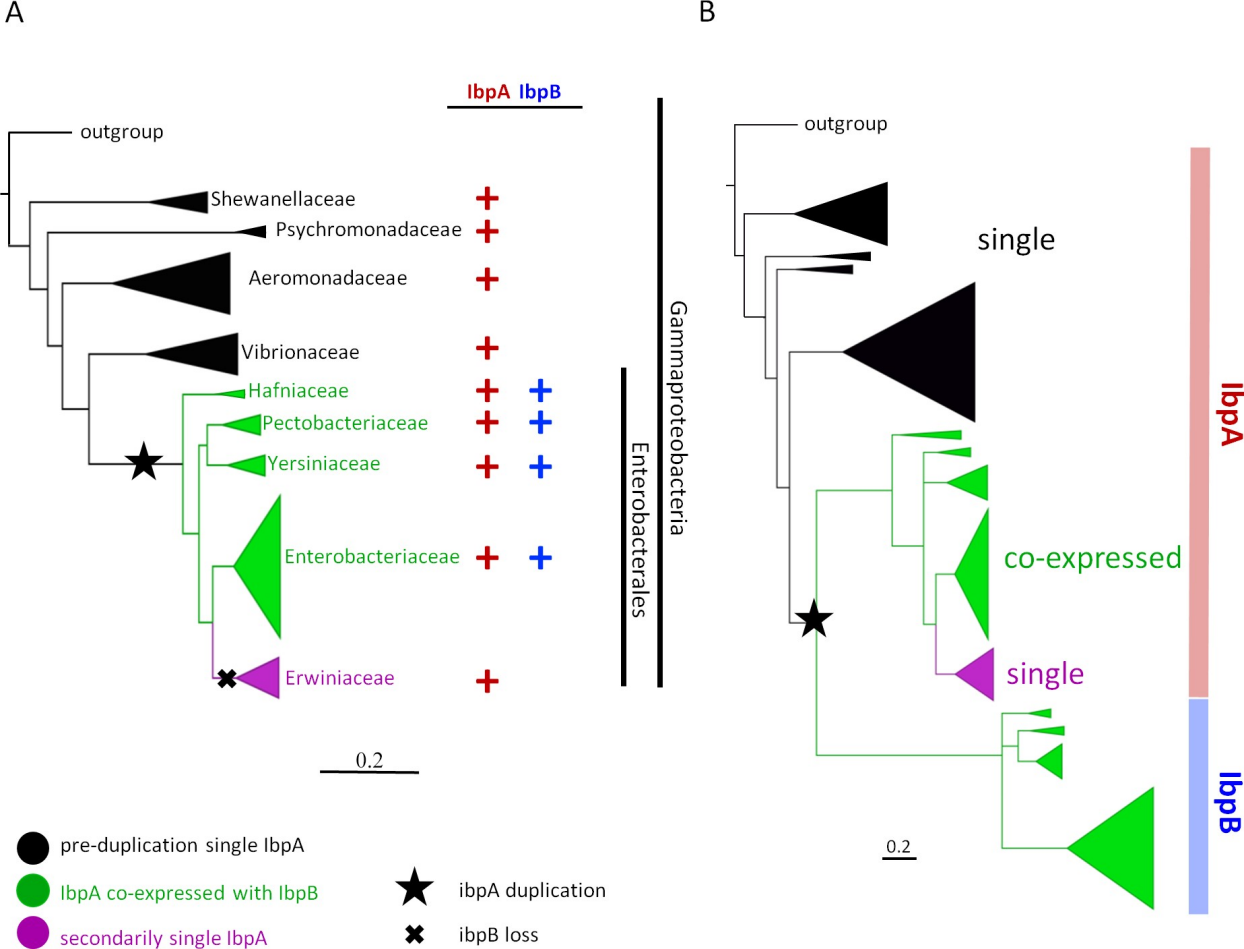
Evolutionary history of sHsps in γ-proteobacteria. (A) The distribution of sHsp genes (*ibpA* red; *ibpB* blue) at the species phylogeny of γ-proteobacteria (calculated with a supermatrix of 1500 orthologs from 50 γ-proteobacteria genomes using Maximum Likelihood method with GTR+GAMMA model) indicates the presence of a single copy *ibpA* (black) in γ-proteobacteria before the speciation of *Enterobacterales*, both *ibpA* and *ibpB* (green) are co-expressed in the subset of *Enterobacterales* (*Hafniaceae*, *Pectobacteriaceae*, *Yersiniaceae*, *Enterobacteriaceae*), and *ibpB* is absent in *Erwiniaceae* (purple). Based on parsimony two evolutionary events in γ-proteobacteria were inferred, the duplication of *ibpA* at the base of *Enterobacterales* (star) and loss of *ibpB* in *Erwiniacea* (cross). (B) The evolutionary history of sHsps (IbpA red; IbpB blue) in γ-proteobacteria, calculated with Maximum Likelihood method with CAT+I+LG model, indicates the presence single duplication event at the base of *Enterobacterales*, resulting in a presence of both IbpA and IbpB in *Hafniaceae*, *Pectobacteriaceae*, *Yersiniaceae*, *Enterobacteriaceae* (green). In all these lineages *ibpB* evolves faster than *ibpA*, but no *ibpB* is present in *Erwiniacea* (purple). Scale bar indicates number of substitution per site.

While many y-proteobacteria possess only one sHsps gene, *E*. *coli* possesses two genes coding sHsps. This suggests that this chaperone system might have been shaped by a gene loss or gene duplication event relatively recently in the evolutionary history of bacteria. To gain insight into how the bacterial protein quality control system has been enriched with another small heat shock protein, we carried out evolutionary analysis of y-proteobacteria and their sHsps. Since the internal phylogeny of y-proteobacteria was not fully resolved, and the position of one of the key phyla, *Erwiniaceae*, remained debated [34–37], we improved on previous phylogenetic analyses by fully sampling 50 y-proteobacteria genomes and obtained a tree of γ-proteobacteria with strong statistical support (all nodes with at least 99% bootstrap support) (Fig 1A, S1 Fig). Similarly to the recent analysis [38], *Erwiniaceae* (the family of *Erwinia amylovora*) and *Enterobacteriacea* (the family of *E*. *coli*) have been reconstructed as sister groups within *Enterobacterales* (the taxonomic subgroup of γ-proteobacteria). We found that *Enterobacterales*, with the exception of *Erwiniaceae*, possess two sHsps (IbpA and IbpB) in contrast to other γ-proteobacteria taxa (*Vibrionaceae*, *Aeromonadaceae*, *Psychomonadaceae*, *Shewanellaceae*), which possess only one sHsp (Fig 1A, S2 Fig). To further investigate sequence evolution, we reconstructed the phylogeny of sHsps. We inferred that there had been a single *ibpA* gene in basal γ-proteobacteria species, which underwent duplication in an ancestor of *Enterobacterales*, resulting in the emergence of an operon comprising *ibpA* and *ibpB* (Fig 1, S2 Fig). We noticed that after the duplication event, *ibpB* sequences evolved faster than *ibpA* (Fig 1B, S2 Fig), suggesting that IbpB protein is functionally divergent from IbpA. Additionally, we found that *ibpB* has been lost in the *Erwiniaceae* clade, and only post-duplication single *ibpA* is present there (Fig 1).

Previous *in vitro* studies of the *E*. *coli* system show that IbpB, contrary to IbpA, does not form sHsp-substrate assemblies [23]. Why, then, the second copy of sHsp gene has been maintained in the majority of the families within the *Enterobacterales* order? To understand the functional divergence of IbpA and IbpB in comparison with single small heat shock protein systems, we used the inferred evolutionary history of sHsps genes in γ-proteobacteria to select model protein representatives for an sHsp that has always been single (IbpA_*Vh*_ from *Vibrio harveyi*), post-duplication IbpA and IbpB (IbpA_*Ec*_ and IbpB_*Ec*_ from *E*. *coli*) and post-duplication secondarily single IbpA (IbpA_*Ea*_ from *Erwinia amylovora*, where IbpB has been lost). All selected bacteria live in a similar range of temperatures, thus one may expect that respective sHsps will be active within a similar range of temperatures.

### IbpA proteins, in contrast to IbpB, efficiently form sHsps-substrate assemblies

**Fig 2:**
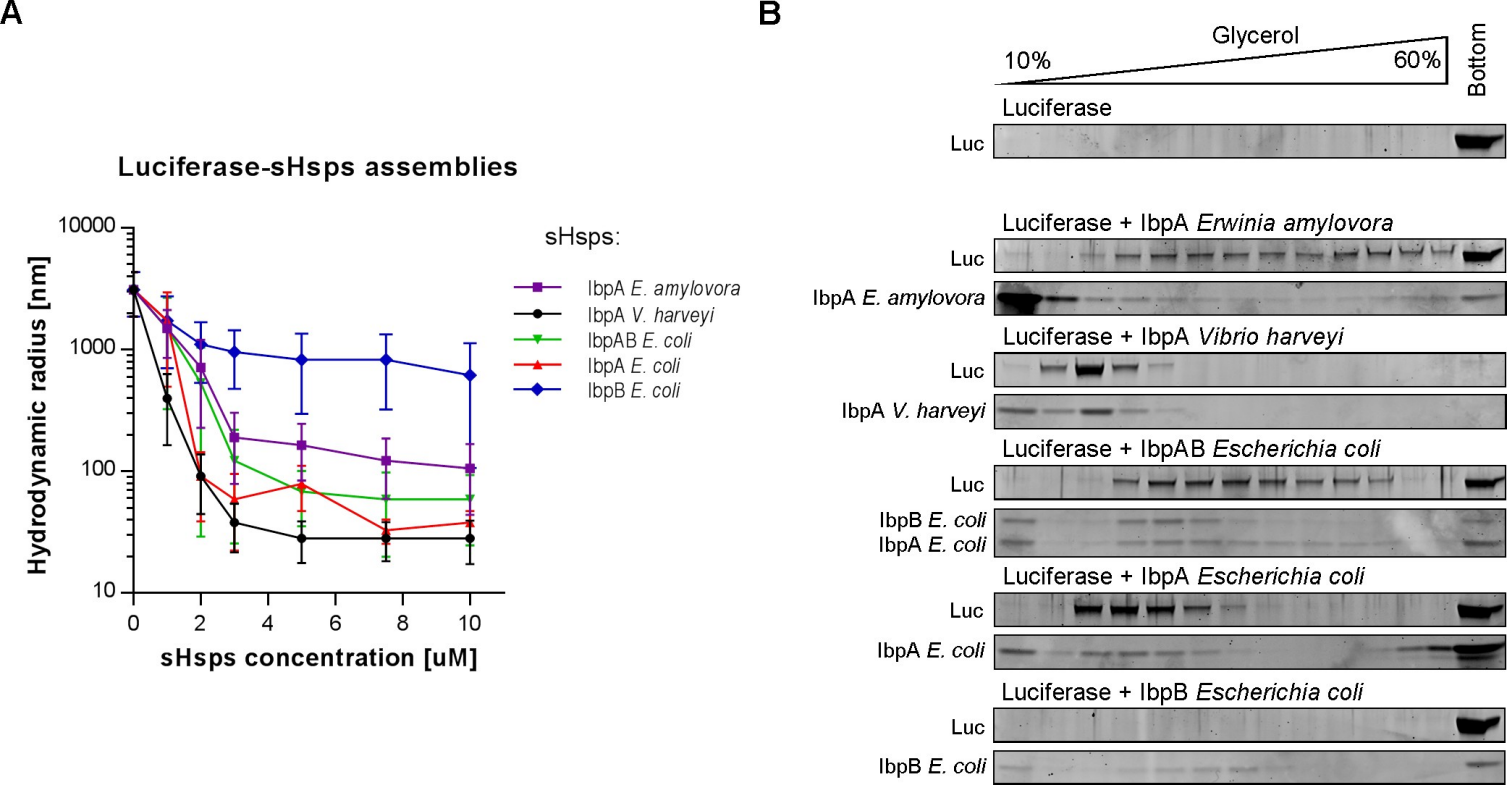
sHsps differ in ability to form assemblies with substrate. (A) DLS measurements of sHsps-luciferase assemblies. Average hydrodynamic radius (±SD) of the most occupied peak (min. 80% of total volume) from DLS size distributions of sHsps-luciferase assemblies. sHsps-luciferase assemblies were prepared as follows: luciferase at fixed 1.5 μM concentration in the presence of sHsps (0 to 10 μM as depicted) was denatured at 44°C for 10 min. When IbpA_*Ec*_ and IbpB_*Ec*_ were tested together (IbpAB_*Ec*_), the 1:2 stoichiometry was used (e.g. 10 μM IbpAB_*Ec*_ is 3.33 μM IbpA_*Ec*_ and 6.67 μM IbpB_*Ec*_). (B) Sedimentation profiles of sHsps-luciferase assemblies. 1 μM luciferase was denatured at 44°C for 10 min in the presence of 5 μM sHsps and subjected to sedimentation in glycerol gradient. Fractions were collected from the top and analysed by SDS-PAGE followed by Oriole staining.

Encaging aggregating proteins in the assemblies is a hallmark of sHsps function in the protein quality control network. To investigate the sHsps’ ability to form the assemblies, we thermally denatured firefly luciferase in the presence of increasing concentration of sHsps and measured size distributions of the resulting assemblies with dynamic light scattering (DLS) (Fig 2A). The two-sHsp system IbpAB_*Ec*_ (IbpA_*Ec*_ and IbpB_*Ec*_), as well as the both single-sHsp systems, IbpA_*Vh*_ and IbpA_*Ea*_, produced at least 1 order of magnitude smaller assemblies (up to 2 orders of magnitude in case of IbpA_*Vh*_) as compared to luciferase aggregated alone. Consistent with the previous studies, IbpA_*Ec*_ in the absence of IbpB_*Ec*_ formed small (<100 nm hydrodynamic radius) assemblies, whereas IbpB_*Ec*_ alone was substantially less effective in this assay (Fig 2A)[23]. Additionally, to check if the differences between the assembly-forming properties of IbpA_*Ec*_ and IbpB_*Ec*_ are not limited to luciferase, we used two other substrates: citrate synthase (CS) and malate dehydrogenase (MDH). We observed that IbpA_*Ec*_ alone is enough to produce small assemblies for luciferase and CS, but to achieve that for MDH, both IbpA_*Ec*_ and IbpB_*Ec*_ were required (S3 Fig). In contrast, IbpB alone was inefficient in the assembly formation, as high level of light scattering was observed with each tested substrate (S3 Fig).

As the sHsp oligomers that are not in complex with a substrate may interfere with the DLS measurement, we decided to further evaluate the assembly-forming properties of analysed sHsps using sedimentation in glycerol gradient. This allowed for easy separation of the sHsps-substrate assemblies that localize in the middle of the gradient from unbound sHsps localized at the top and big aggregates at the bottom of the gradient. For the individual systems, we observed slight differences in the sedimentation profiles, with the smaller size assemblies (e.g. IbpA_*Vh*_-luciferase) localized closer to the top of the gradient and larger species (e.g. IbpA_*Ea*_-luciferase) closer to the bottom (Fig 2B). Additionally, although some of IbpB_*Ec*_ was present in the middle of the gradient, it was not associated with any detectable amount of luciferase (Fig 2B), which indicates the inability of IbpB_*Ec*_ alone to form stable small–size assemblies. All the obtained glycerol gradient profiles are in agreement with the DLS measurements (Fig 2A).

**Fig 3:**
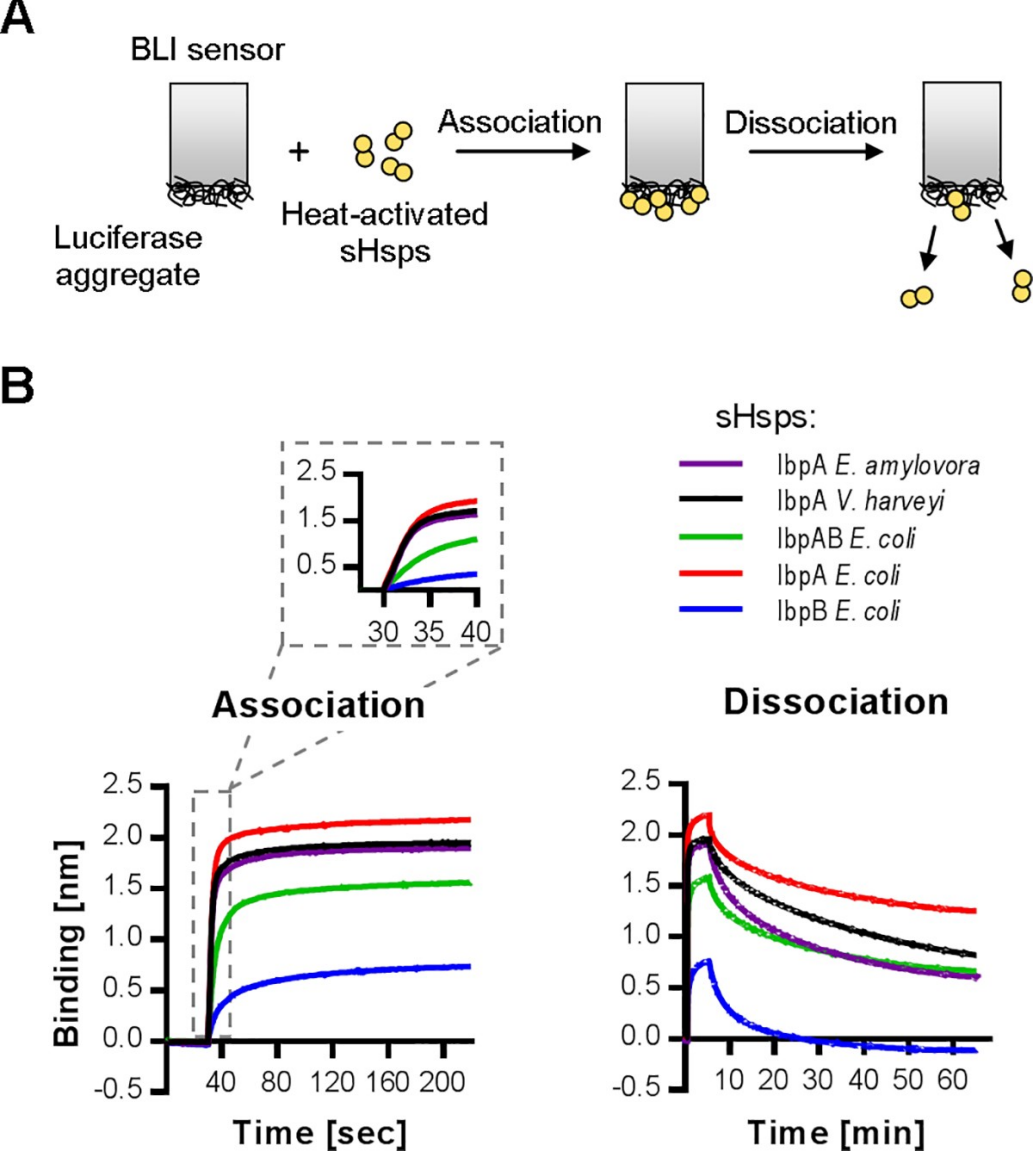
IbpA small heat shock proteins stably bind aggregated substrates. (A) Experimental scheme. Heat-activated sHsps (44°C, 5 min) were applied onto BLI sensor with immobilized aggregated luciferase and subsequently dissociated in the plain buffer. (B) Association and dissociation curves. sHsps were used in 5 μM concentration, IbpAB_*Ec*_ stoichiometry as in Fig 2A.

Formation of the assemblies is driven by sHsps’ ability to interact with an unfolded substrate. The bacterial sHsps form large, undefined oligomers and their substrates are heterogeneous, unfolded polypeptides, which makes assessment of this interaction challenging. We approached this problem with biolayer interferometry (BLI), using aggregated luciferase immobilized on the surface of a BLI sensor as a substrate for heat-activated sHsps (Fig 3A). Changes in the thickness of the protein layer due to the association and dissociation of sHsps allow for comparison of the sHsps’ ability to interact with the aggregated substrate. In this assay, all the sHsps bind to the sensor within single seconds. Their dissociation requires much longer time (tens of minutes) (Fig 3B), which is consistent with their widely recognized holdase activity. The most efficient binder is IbpA_*Ec*_, closely followed by the single-sHsp systems: IbpA_*Ea*_ and IbpA_*Vh*_, which bind equally fast but generate slightly thinner protein layers (Fig 3B). The two-protein system IbpAB_*Ec*_ binds less effectively both in terms of the binding rate and increase in the protein thickness. Dissociation of all the sHsps is comparable, the complex with IbpA_*Ec*_ being the most stable, as most of it remained on the sensor even after 60 min of washing (Fig 3B). IbpB_*Ec*_ is clearly the least efficient binder in this assay. It binds with the slowest kinetics and generates substantially smaller increase (one third) in the thickness of the protein layer. It is also the only sHsp that completely dissociates from the sensor (Fig 3B). This data is in good agreement with previously analysed assembly-forming sHsps activities (Fig 2).

**Fig 4:**
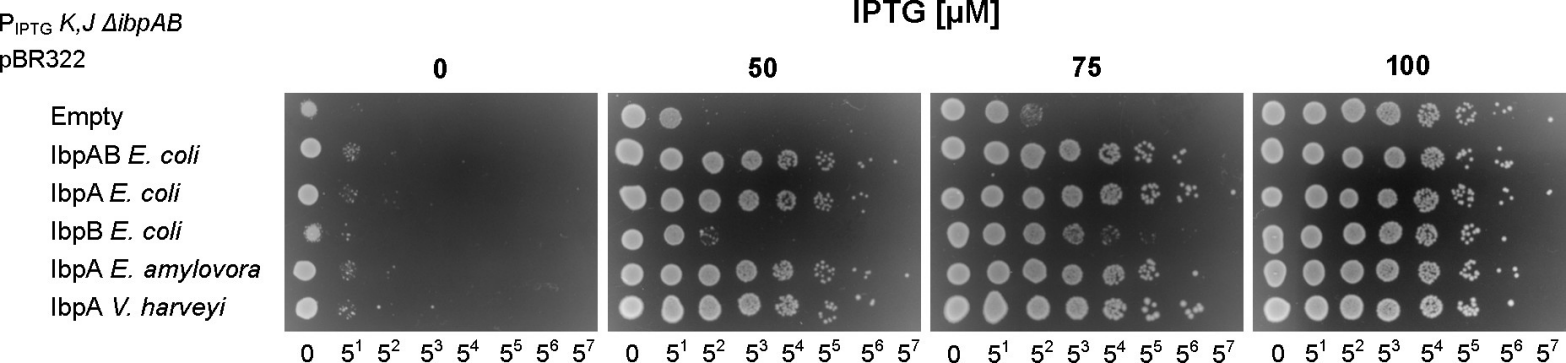
IbpA-like but not IbpB small heat shock proteins complement Δ*ibpAB* phenotype in *E*. *coli* with reduced DnaK levels. *E*. *coli* P_IPTG_ *dnaKJ* strains expressing indicated sHsps were grown at 30°C in presence of 1 mM IPTG until late logarithmic phase. Serial dilutions of bacterial cultures were plated on LA medium supplemented with indicated concentration of IPTG and grown in 37°C overnight.

Next, we tested whether the ability of sHsps to form assemblies *in vitro* corresponds to the phenotypes of the *E*. *coli* cells carrying the respective *sHsps* genes. For this analysis we used an *E*. *coli* strain developed by Mogk and colleagues [4], in which the *ibpAB* operon has been deleted and *dnaK* and *dnaJ* are under the *pLac* promoter, which allows to regulate their expression (S4 Fig). As reported [4], in such *E*. *coli* strain, when DnaK and DnaJ are downregulated, the formation of colonies depends on the presence of IbpAB. We cloned the *ibpAB* operon in its native version or carrying the nonsense codon either in *ibpA* (for IbpB expression), or in *ibpB* (for IbpA expression) into pBR322 plasmid. For heterologous sHsps expression, *ibpA*_*Ea*_ or *ibpA*_*Vh*_ were cloned instead of *ibpAB*_*Ec*._ In consequence, all the cloned sHsps genes were under the same heat shock promoter and produced the respective sHsps at the heat shock conditions, although the plasmid-borne IbpA_*Vh*_ and IbpB_*Ec*_ levels were higher compared to the IbpAB_*Ec*_, IbpA_*Ec*_ and *IbpA*_*Ea*_ levels (S5 Fig). In this experiment, the expression of either IbpAB_*Ec*_, IbpA_*Ec*_ alone, IbpA_*Ea*_ or IbpA_*Vh*_ rescued the ability of *E*. *coli* to form colonies in contrast to the strain carrying the empty plasmid (Fig 4). The expression of IbpB alone resulted in only very limited increase in the formation of colonies (Fig 4), even though IbpB was expressed at high level. Thus, there is a very good correspondence between the growth of *E*. *coli* expressing the specific sHsps and the ability of these sHsps to form assemblies with the substrates. Additionally, this experiment showed that IbpA_*Ea*_ and IbpA_*Vh*_ are functional in *E*. *coli*.

Summing up, we show that all the IbpA sHsps, as well as the two-protein IbpAB_*Ec*_ system are similarly functional in substrate binding and assembly formation, which translates into the rescue of growth of the chaperone-deficient cells. On the other hand, IbpB_*Ec*_ alone seems to be irrelevant for assembly formation, which is in line with the previous reports, showing that IbpB does not impact the morphology of protein aggregates. However, the same studies indicate that IbpB has remained important for efficient Hsp70-Hsp100-mediated substrate disaggregation and refolding [23, 24].

Based on the distinct behaviour of IbpA and IbpB, we infer that, after duplication in the common ancestor of *Enterobacterales*, sHsps have functionally diverged in a way that one of the paralogs, IbpA, has retained the canonical holdase activity, while the other paralog, IbpB, has lost this activity but contributes to the effective disaggregation at a different stage of the process.

### IbpAB-substrate assemblies require less Hsp70 than IbpA-substrate assemblies for efficient refolding

sHsps-substrate assemblies serve as a storage for denatured polypeptides that prevents their further aggregation until the conditions become favourable for the Hsp70-Hsp100 machinery and allow for active protein refolding. To initiate the refolding, sHsps are outcompeted from the assembly outer shell by Hsp70. This phase has been suggested to be the rate-limiting step in substrate refolding and it strongly depends on the Hsp70 level [9]. Therefore, we decided to analyse the requirements for Hsp70 in protein refolding from assemblies formed in the presence of different sHsps. Based on our previous work with the IbpAB_*Ec*_-luciferase assemblies [9], we chose two concentrations of the Hsp70 system: limiting KJE (0.7 μM DnaK, 0.28 μM DnaJ, 0.21 μM GrpE) and saturating KJE (3.5 μM DnaK, 1.4 μM DnaJ, 1.05 μM GrpE). We assessed the efficiency of luciferase reactivation that had been heat-aggregated with: IbpA_*Vh*_, IbpA_*Ea*_, IbpA_*Ec*_, IbpB_*Ec*_ or IbpAB_*Ec*_. For refolding of substrates from the assemblies formed by IbpA from *E*. *amylovora* or *V*. *harveyi*, the Hsp70 system from *E*. *coli* was used since both IbpA_*Ea*_ and IbpA_*Vh*_ were functional in the *in vivo* complementation experiment (Fig 4).

**Fig 5:**
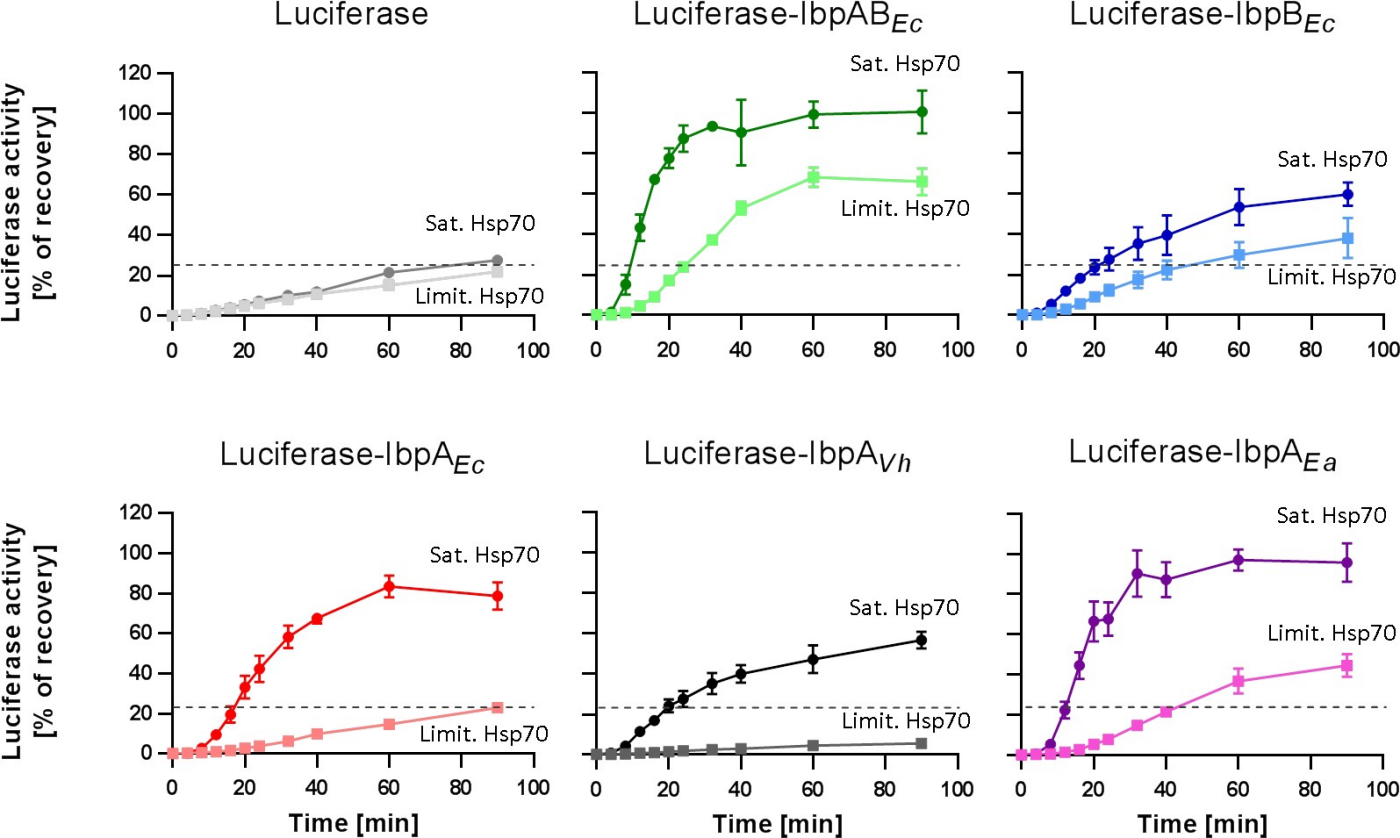
Refolding potential of sHsps-substrate assemblies. Luciferase (1.5 μM) was denatured in the presence of 10 μM sHsps (IbpAB_*Ec*_ stoichiometry as in Fig 2A), diluted to 60 nM and refolded at limiting (DnaK 0.7 μM; DnaJ 0.28 μM; GrpE 0.21 μM) or 5x higher saturating (DnaK 3.5 μM; DnaJ 1.4 μM; GrpE 1.05 μM) Hsp70 machinery concentrations and ClpB at 2 μM concentration. Data are the mean ± SD of three independent experiments.

At the limiting Hsp70 system concentration, sHsps-luciferase assemblies containing IbpA_*Vh*_ or IbpA_*Ec*_ were refolded at a similar or lower rate as compared to refolding of luciferase from aggregates formed in the absence of any sHsps (Fig 5). Thus, no positive effect but rather inhibition of refolding was observed. At the same conditions, luciferase denatured with IbpA_*Ea*_ or IbpB was refolded only slightly more effectively than luciferase from aggregates lacking sHsps. The highest refolding efficiency was observed for the IbpAB_*Ec*_-luciferase assemblies (Fig 5). Once similar experiments were performed in the presence of the saturating Hsp70 concentration, a substantial increase in refolding efficiency was observed for assemblies formed in the presence of each IbpA protein (IbpA_*Vh*_, IbpA_*Ea*_ or IbpA_*Ec*_) (Fig 5). In contrast, increase of the Hsp70 level did not result in more efficient luciferase refolding from aggregates formed in the absence of any sHsps (Fig 5). This shows that Hsp70 is a limiting factor required for efficient refolding of substrates from the assemblies formed in the presence of any IbpA protein. In the two-protein system, however, the presence of IbpB alongside its IbpA partner during the assembly formation substantially lowers the demand for Hsp70 in refolding.

To assess if the observed phenomenon is *E*. *coli*-specific or of a more general nature, we decided to perform additional experiments involving another two-protein sHsp system. We selected the system from *Cedecea neteri* (IbpA_*Cn*_ and IbpB_*Cn*_), a member of *Enterobacteriaceae* distantly related to *E*. *coli* (S2 Fig). *C*. *neteri* is a free-living bacterium that can be pathogenic to human, causing bacteraemia in heart disease patients. Alongside IbpAB_*Cn*_, we included in our studies an additional single-sHsp from *Aeromonas hydrophila* (IbpA_*Ah*_), an opportunistic human pathogen that is also considered a major fish and amphibian pathogen. Both species are included in our phylogenetic analysis (S2 Fig).

**Fig 6:**
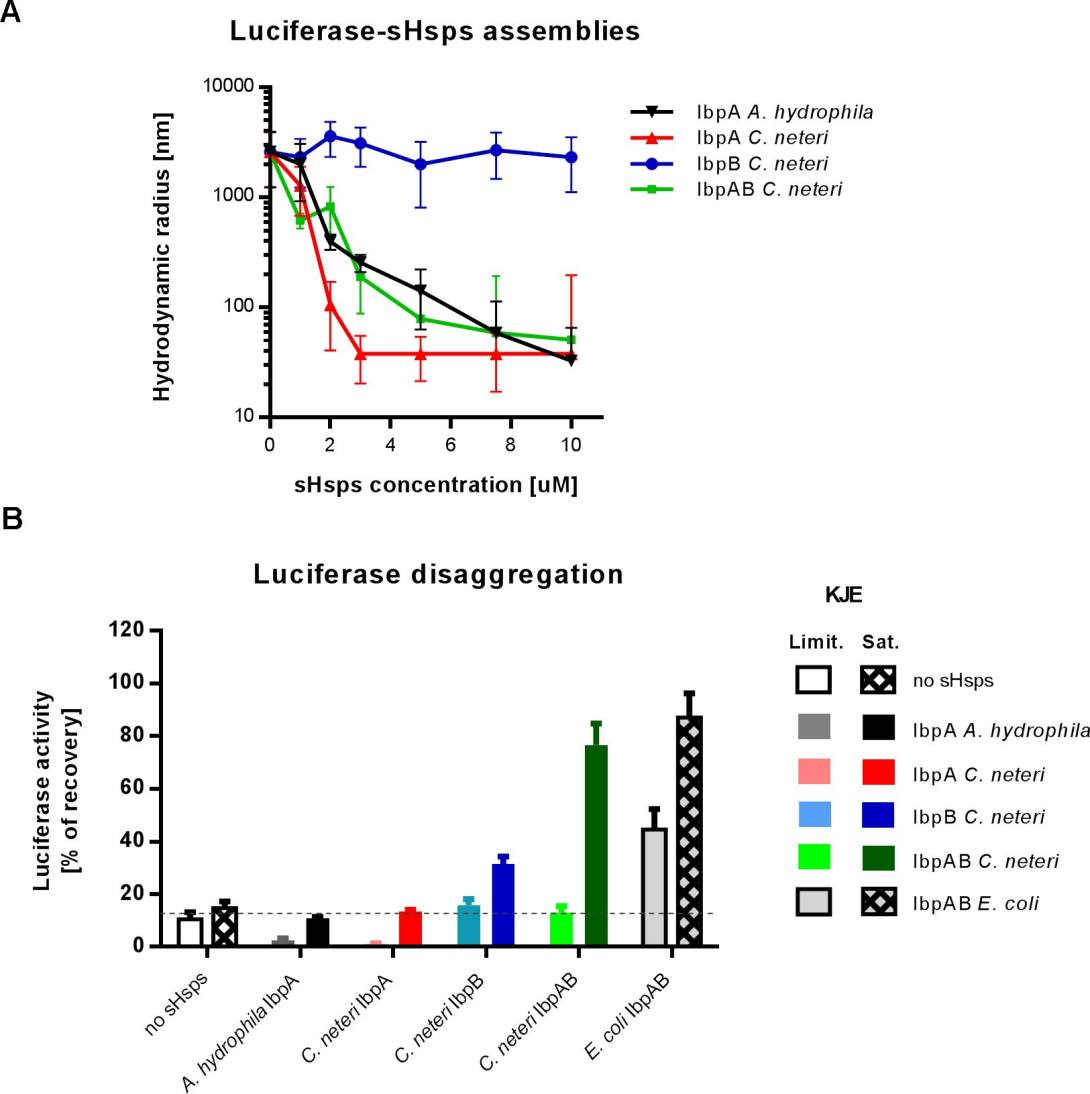
IbpBs do not form assemblies with the substrate but increase efficient of substrate refolding. (A) DLS measurements of sHsps-luciferase assemblies. Average hydrodynamic radius (±SD) of the most occupied peak (min. 80% of total volume) from DLS size distributions of sHsps-luciferase assemblies is presented. Experimental conditions as in Fig 2A. (B) Refolding potential of sHsps-substrate assemblies. Luciferase (1.5 μM) was denatured in the presence of 10 μM sHsps (IbpAB_*Ec*_ and IbpAB_*Cn*_ stoichiometry as in Fig 2A), diluted to 60 nM and refolded at limiting (DnaK 0.7 μM; DnaJ 0.28 μM; GrpE 0.21 μM) or 5x higher saturating (DnaK 3.5 μM; DnaJ 1.4 μM; GrpE 1.05 μM) Hsp70 machinery concentrations and ClpB at 2 μM concentration (as in Fig 5) for 40 min.

We repeated the key experiments with purified sHsps from the selected species. We tested their ability to form small-size assemblies with the aggregating substrate and the consequences of their presence in the assemblies on the efficiency of refolding by the Hsp70-Hsp100 system. Both single IbpA from *C*. *neteri* and *A*. *hydrophila*, similarly to the other tested IbpA proteins (Fig 2A), efficiently generate small-size assemblies (<100 nm hydrodynamic radius) (Fig 6A). Similarly to IbpB_*Ec*_, IbpB_*Cn*_ is unable to form such small-size assemblies. Consistently with the previous refolding experiments (Fig 5), all the IbpA proteins within the assemblies inhibit substrate refolding (Fig 6B). However, neither *A*. *hydrophila* nor *C*. *neteri* IbpA-dependent inhibition was overcome at the saturating KJE concentration (Fig 6B). The activity of IbpB_*Cn*_ alone resembles that of IbpB_*Ec*_, causing limited improvement in refolding (Figs 5 and 6B). Finally, when IbpB_*Cn*_ was present in the assemblies alongside IbpA_*Cn*_, we observed potent refolding when KJE was used at the saturating concentration (Fig 6B).

The features of the *C*. *neteri* IbpAB system are consistent with the *E*. *coli* IbpAB system. Although the assemblies containing IbpAB_*Cn*_ require higher KJE concentration for efficient refolding compared to IbpAB_*Ec*_, the presence of IbpB_*Cn*_, similarly to the *E*. *coli* system, allows for effective refolding, which is inhibited in the presence of IbpA_*Cn*_.

### IbpB reduces the demand for Hsp70 to outcompete IbpA from aggregated substrate

**Fig 7:**
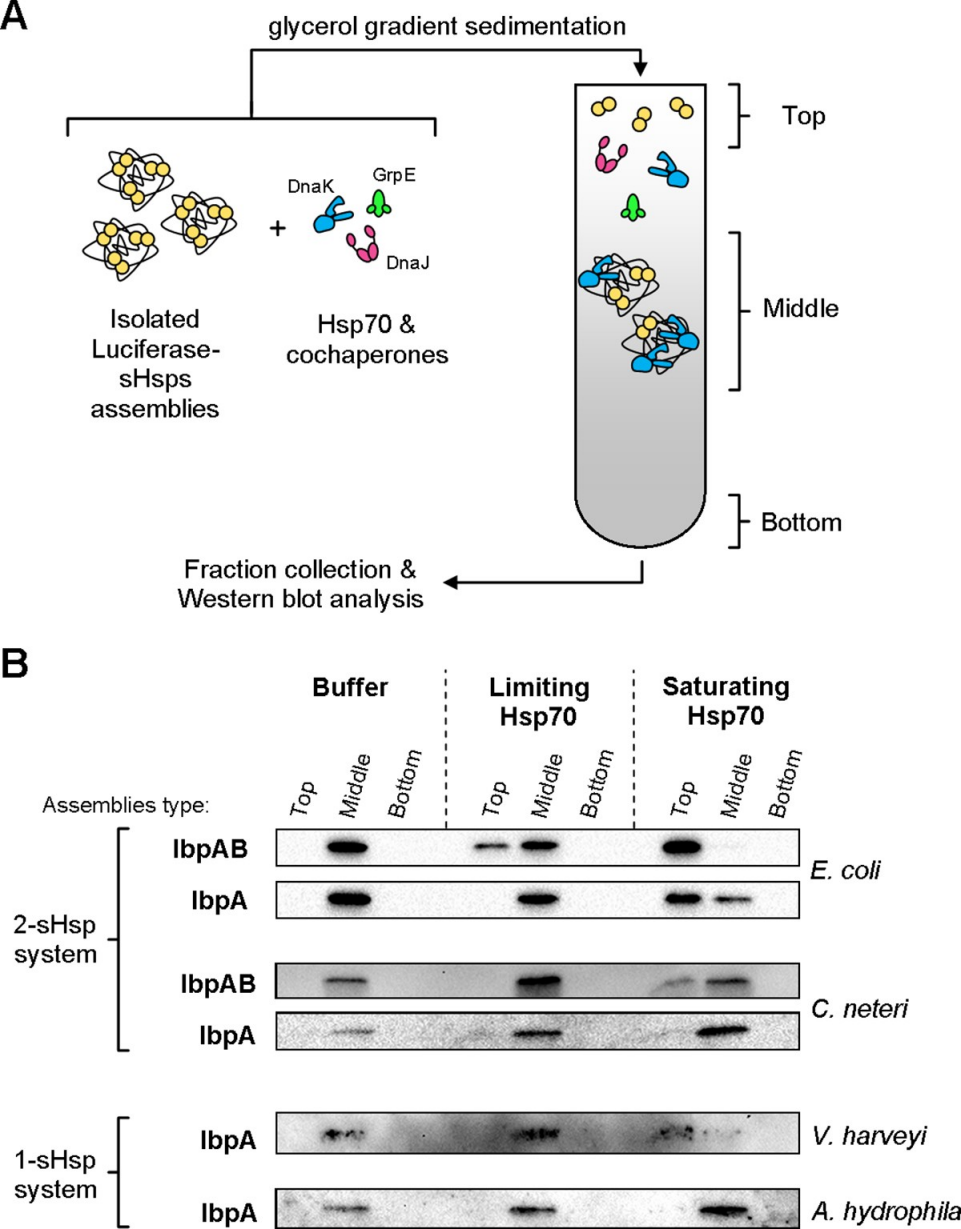
IbpB presence in IbpAB–substrate assemblies allows for efficient Hsp70-dependent dissociation of IbpA from assemblies. (A) Experimental scheme. Purified IbpA- and IbpAB-luciferase assemblies were incubated with indicated components and resubjected to glycerol gradient sedimentation. (B) Isolated sHsps-luciferase assemblies were incubated with buffer or limiting (DnaK 0.7 μM; DnaJ 0.28 μM; GrpE 0.21 μM) or saturating (DnaK 3.5 μM; DnaJ 1.4 μM; GrpE 1.05 μM) Hsp70 machinery concentration followed by glycerol gradient sedimentation. Fractions were collected from the top, pooled (top—fractions containing free sHsps; middle—fractions containing sHsps-luciferase assemblies; bottom–material recovered from the bottom of centrifugation tube) and analysed by Western blot with IbpA antibodies following SDS-PAGE. The experiments were repeated at least twice.

Hsp70-Hsp100-mediated disaggregation and refolding consists of several steps. Firstly, Hsp70 recognizes and binds to aggregates, which requires the removal of sHsps. Secondly, Hsp70 binding determines the efficiency of Hsp100 recruitment to the aggregates and its activation for forceful polypeptide extraction. Finally, Hsp70 may be involved in polypeptide folding downstream of Hsp100. This complex process leads to properly folded and active proteins. Thus, the activity of the refolded substrate gives an information of the overall recovery efficiency. To analyse the sHsps-Hsp70 interplay exclusively at the aggregate recognition and binding by Hsp70 step (where sHsps-mediated inhibition occurs), we performed sedimentation analysis. We wanted to investigate if IbpB presence allows for more efficient IbpA dissociation upon DnaK binding to assemblies. We isolated assemblies formed by luciferase denatured in the presence of IbpA or IbpAB *via* sedimentation in glycerol gradient (S6 Fig). Subsequently, we incubated the purified assemblies at the limiting or saturating KJE concentration or in the buffer only, and we tested them for integrity by subjecting to a second round of sedimentation (Fig 7A). Pooled fractions containing free sHsps from the top of the gradient, the middle fractions with the assemblies and the bottom fraction (S6 Fig) were analysed with Western blot using antibodies against IbpA (Fig 7A). In this experiment, the limiting Hsp70 system facilitates dissociation of substantial amount of IbpA_*Ec*_ from the IbpAB_*Ec*_-containing assemblies (Fig 7B). In the absence of IbpB_*Ec*_, dissociation of IbpA_*Ec*_ from the assemblies requires high Hsp70 concentration, although the process is still less effective than for IbpAB_*Ec*_ (Fig 7B). The analysis of IbpAB_*Cn*_ assemblies revealed that IbpA_*Cn*_ was released only at high Hsp70 concentration. In the absence of IbpB_*Cn*_, IbpA_*Cn*_ was no longer released and remained in assemblies (middle) fraction. The analysis of the single-sHsps systems showed that IbpA_*Vh*_ dissociated only at high Hsp70 concentration while IbpA_*Ah*_ remained associated with assemblies at all conditions tested.

We additionally performed a similar experiment with IbpA_*Ec*_ and IbpB_*Ec*_ using another substrate, Citrate Synthase. Similarly, to the luciferase assemblies, IbpB enhanced dissociation of IbpA from the assemblies both at the limiting and saturating concentrations of Hsp70 (S7 Fig).

These data correlate well with the refolding experiments, and indicate that protein refolding strongly depends on the sHsps dissociation from the assemblies. Moreover, in comparison to IbpA alone, the presence of IbpB in both of the analysed two-sHsp systems allows for sHsps removal at lower Hsp70 concentration. In consequence, the presence of IbpB provides more efficient disaggregation and refolding at limited availability of the Hsp70 chaperone.

**Fig 8:**
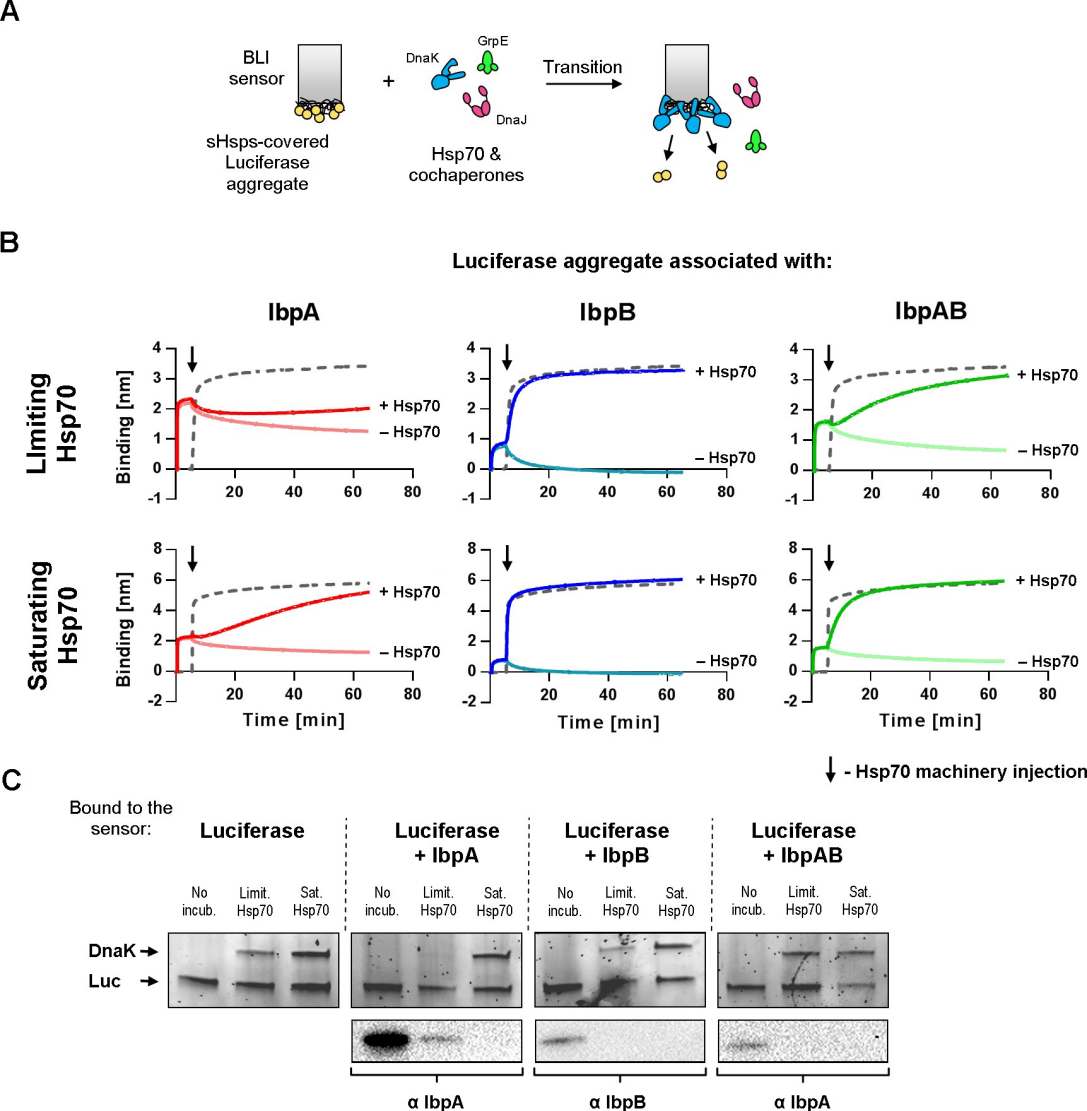
Aggregate-bound sHsps differently inhibit Hsp70 binding. (A) Experimental scheme. (B) Aggregated luciferase immobilized on the BLI sensor was associated with sHsps as in Fig 3B and subsequently introduced to buffer containing limiting (DnaK 0.7 μM; DnaJ 0.28 μM; GrpE 0.21 μM) (upper panels) or saturating (DnaK 3.5 μM; DnaJ 1.4 μM; GrpE 1.05 μM) (lower panels) Hsp70 machinery concentrations (dark traces). Light traces represent spontaneous sHsps dissociation curves (as shown in Fig 3B), grey dashed traces show Hsp70 machinery binding to immobilized luciferase aggregates in the absence of any sHsps. (C) Sensor-bound proteins were analysed before and after Hsp70 machinery action with SDS-PAGE followed by Western blot using IbpA or IbpB antibodies and Oriole staining for DnaK and luciferase. No incubation lanes refer to proteins associated with the sensor prior to incubation with either limiting or saturating Hsp70 concentration. The experiments presented in panel B were repeated at least twice, supporting SDS-PAGE (Oriole stained) and Western blot analyses (panel C) were performed once.

To gain insight into the kinetics of the exchange between sHsps and Hsp70 on the assemblies, we modified the BLI experiment in a way that a sensor with aggregated luciferase covered with heat-activated sHsps was introduced into a buffer containing either the limiting or saturating KJE concentrations (DnaK, DnaJ, GrpE) (Fig 8A). Although with BLI we cannot distinguish between the proteins bound to the sensor, we took advantage of the differences in the kinetics of binding and the thickness of the protein layer associated with sHsps or Hsp70 binding to the sensor (Fig 8B). We inferred that the increase in the signal above the sHsps binding plateau upon addition of the Hsp70 system can be interpreted as Hsp70 binding. Additionally, to confirm Hsp70 association and sHsps dissociation, we analysed the proteins bound to the sensor before and after 60 minutes of incubation in the presence of the Hsp70 system, For protein detection, we used SDS-PAGE followed by fluorescent staining (for DnaK) and Western Blot (for sHsps) (Fig 8C).

The analysis of the binding curves obtained at limiting KJE concentration clearly shows that IbpA_*Ec*_ alone associated with the aggregates substantially inhibits the binding of DnaK to the sensor (Fig 8B). In contrast, IbpB_*Ec*_ alone has no negative effect on DnaK binding to the sensor (Fig 8B). Association of IbpAB_*Ec*_ with aggregates exerts a moderate effect, causing the delay in binding of DnaK to the sensor (Fig 8B). The binding curves data are in agreement with the SDS-PAGE analysis of proteins associated with the sensor after 60 minutes of incubation with Hsp70 (Fig 8C). No DnaK, but IbpA_*Ec*_, was detected on the sensor when IbpA_*Ec*_ alone was initially associated with aggregates. In contrast, when IbpB_*Ec*_, or IbpAB_*Ec*_ or no sHsp had been originally bound to aggregates, DnaK was found associated after sensor incubation with limiting KJE concentration. When the same experiment was performed at the saturating Hsp70 concentration, we observed much shorter delay in DnaK binding to the IbpAB_*Ec*_-shielded aggregates. Also, slow binding kinetics of DnaK was obtained for the IbpA_*Ec*_-shielded aggregates (Fig 8B). These results were further confirmed by the SDS-PAGE analysis of proteins associated with the sensor (Fig 8C). In contrast to the limiting Hsp70 conditions, DnaK but not IbpA_*Ec*_, was found to be associated with aggregates that initially were associated with IbpA_*Ec*_. This data show that the high demand for the Hsp70 system observed for IbpA_Ec_-substrate assemblies (Fig 5) is due to higher IbpA_Ec_ resistance to be outcompeted by DnaK, which is relieved by IbpB_Ec_ (Figs 7B and 8B and 8C).

Both the sedimentation experiments (Fig 7, S7 Fig) and the BLI analysis (Fig 8) showed that IbpB presence in the assemblies allow for more efficient IbpA dissociation in the presence of Hsp70. This in turn allows for more effective DnaK association with the assemblies, which promotes further steps of substrate extraction and refolding.

### *In vivo*, IbpB is directed to the aggregated protein fraction by IbpA, where it ensures IbpA release during disaggregation

**Fig 9:**
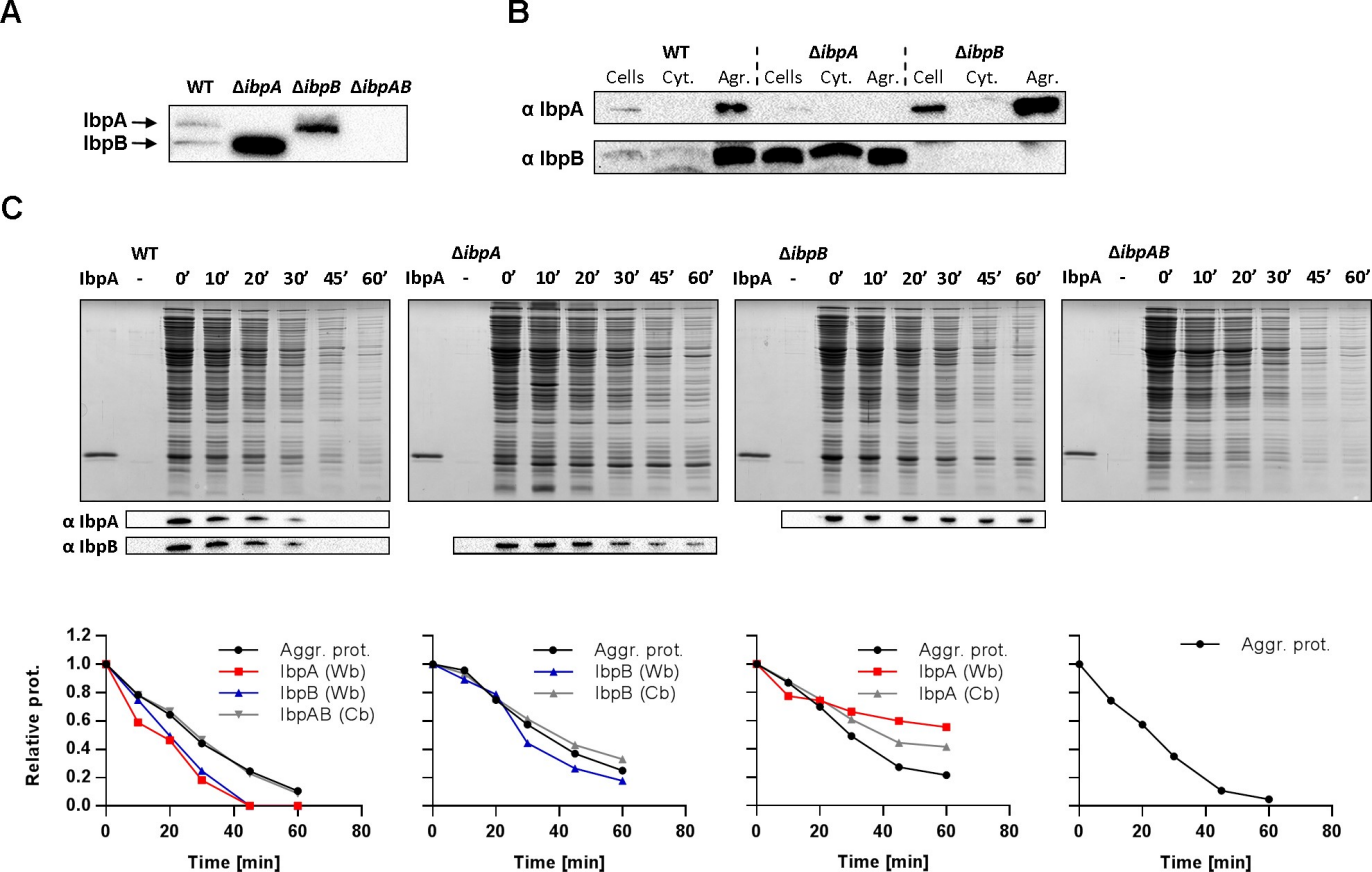
IbpB modulates the association of IbpA with aggregates *in vivo*. (A) Expression of IbpA and IbpB in constructed strains. *E*. *coli* MC4100 WT, Δ*ibpA*, Δ*ibpB* and Δ*ibpAB* cultures grown in 30°C were subjected to heat shock (42°C, 10 min and 48°C, 5 min) and analysed with SDS-PAGE followed by western blot against IbpA and IbpB. (B) Localization of IbpA and IbpB in soluble and aggregated protein fractions. WT, Δ*ibpA* and Δ*ibpB* strains were heat-shocked as in A, followed by isolation of soluble and aggregated protein fractions as described in Methods. Obtained fractions were then analysed by SDS-PAGE and Western blot. (C) IbpB presence allows for IbpA removal from aggregates. Bacterial strains were heat-shocked as in A and allowed to recover at 30°C. Aggregated and soluble protein fractions were isolated from culture aliquots sampled at indicated time points and further analysed by SDS-PAGE Coomassie staining and Western blot using antibodies against IbpA or IbpB. Quantifications of total aggregated proteins (Aggr. prot.) and sHsps (both Western blot -Wb and Coomassie blue -Cb analysis) were plotted against recovery time.

All our *in vitro* experiments indicate that in the analysed sHsp systems of *E*. *coli* and *C*. *neteri* IbpA is responsible for tight binding of aggregating proteins, while IbpB on its own possesses much lower potential of binding aggregating substrates. Nevertheless, IbpB is required for efficient dissociation of sHsps upon Hsp70 binding at the start of refolding. To evaluate *in vivo* our *in vitro* findings, we constructed *E*.*coli* Δ*ibpA* and Δ*ibpB* strains introducing single nonsense mutations on the chromosome using the CRISPR technique. This approach was chosen to minimize the changes in the secondary structure of the mRNA, as *ibpA* and *ibpB* genes are arranged in a single operon and transcribed into polycistronic mRNA. However, as reported previously [39], nonsense mutations in either *ibpA* or *ibpB* resulted in the increase in the cellular level of the remaining sHsp expressed at heat shock conditions as compared to the wild type strain (Fig 9A).

In order to evaluate association of IbpA and IbpB with aggregates, we analysed their presence in soluble and aggregated protein fractions from heat-shocked bacterial cultures (Fig 9B). In the wild type strain both IbpA and IbpB were found mainly in the aggregated protein fraction. In agreement with its high binding potential, IbpA in the Δ*ibpB* strain was exclusively present in the aggregated protein fraction, while IbpB in the Δ*ibpA* strain was detected both in the soluble (approx. 90% of total IbpB) and in the aggregated fraction (approx. 10%) (Fig 9B). Taken together, both *in vivo* and *in vitro* experiments highlight clear differences in IbpA and IbpB aggregate binding potential.

Finally, we wanted to assess if, as observed *in vitro*, IbpB also facilitates dissociation of IbpA from the aggregates *in vivo*. We analysed protein disaggregation process by isolating the aggregated protein fraction from bacterial cultures at different time points during recovery after heat shock. The presence of IbpA and IbpB in this fraction was analysed by Western blot and Coomassie blue staining. In the wild type strain and in all the knock-out mutants, both the proportion of aggregated proteins (approx. 2.8% of the total protein content) and the kinetics of aggregate removal were similar (Fig 9C). In the wild type strain, both IbpA and IbpB were simultaneously removed from the aggregates, slightly faster than aggregated proteins as judged by Western blot. This observation is in agreement with the recently proposed disaggregation mechanism that involves sHsps removal before protein refolding [9]. In Δ*ibpA* strain, IbpB co-disappeared together with aggregates and in Δ*ibpB* strain, IbpA was maintained in the aggregated fraction while disaggregation of other proteins was unaffected (Fig 9C). Thus, *in vivo* IbpB presence is important for IbpA release from the aggregated protein fraction. The absence of IbpB has no effect on the disaggregation process under conditions tested, however we cannot exclude that a small fraction of substrates requires the simultaneous presence of both IbpA and IbpB for efficient disaggregation.

Our *in vivo* studies are in good agreement with the *in vitro* experiments, supporting the hypothesis of IbpA being the strong polypeptide binder and IbpB being the factor allowing easier dissociation of sHsps from aggregates at the initiation of disaggregation.

## Discussion

sHsps act as a first line of defence during proteotoxic stress, interacting with misfolding proteins to modify the aggregation process. The majority of γ-proteobacteria possess only one sHsp (IbpA), while in the last common ancestor of *Enterobacterales*, sHsp gene has undergone duplication. In consequence, the organisms in this order have two sHsps (IbpA and IbpB). Our phylogenetic analysis indicates that the additional sHsp copy (*ibpB*) evolved faster than *ibpA*, suggesting its functional divergence. We confirmed this using biochemical assays, showing that IbpB alone is no longer capable of forming stable assemblies with protein substrates. IbpB, despite having lost its holdase activity, has been maintained as a second sHsp because it has acquired a new function. We show that IbpB cooperates with IbpA in a way that allows for easier release of sHsps from the sHsp-substrate assemblies at the initiation of the Hsp70-Hsp100-dependent protein refolding.

The weak ability of IbpB to associate with aggregating polypeptides suggested by our *in vitro* work is in agreement with previous cellular localization studies, showing that in the absence of IbpA, IbpB is found in cytoplasm [20]. Consistently, our results for the Δ*ibpA* strain show that at heat shock conditions, only a fraction of IbpB is found in the aggregates. In contrast, when IbpA is expressed, the aggregated fraction contains most of the cellular IbpB. Thus, the presence of IbpA efficiently directs IbpB to aggregates. Curiously, the studies of the protein–protein interaction network in *E*. *coli* [40] show that the pool of interacting partners of IbpA is much bigger than that of IbpB (42 and 10, respectively), and that 9 out of 10 partners of IbpB also interact with IbpA [40]. This suggests that IbpB interacts with misfolded substrates *via* IbpA.

The functional cooperation between IbpA and IbpB involves: (*a*) joint formation of assemblies that are more manageable by Hsp70-Hsp100 [9, 23], (*b*) their ability to form mixed complexes *in vitro* [23, 41] and (*c*) mutual influence of each other on their *in vivo* and *in vitro* degradation rates [42, 43]. The ability of IbpA and IbpB to form mixed complexes [23, 41] is probably the key factor determining the formation of the refolding-potent assemblies. However, it is not known what the basic building blocks of such IbpA-IbpB complexes are. The two most possible scenarios involve IbpA and IbpB forming either heterodimers or homodimers that further hetero-oligomerize. In both cases the formation of such complexes results in the incorporation of IbpB molecules, alongside IbpA, into the assemblies during stress. In consequence, the co-introduced IbpB, with low substrate binding potential, decreases the local IbpA-to-substrate ratio and weakens the interaction with substrates. As a consequence, sHsps dissociation from the assemblies is easier which favours a rapid initiation of disaggregation (Figs 7, 8 and S7).

The system composed of the two cooperating sHsps (IbpA and IbpB) appears to be beneficial for the refolding process in comparison to single-sHsp (IbpA) systems. All the analysed IbpA proteins, namely: IbpA from *V*. *harveyi*, *A*. *hydrophila* (pre-duplication), *E*. *amylovora* (lost *ibpB*), *E*. *coli* and *C*. *neteri* (two-sHsp systems), were similarly efficient in the formation of the assemblies with protein substrates. However, in both analysed two-sHsp systems (*E*. *coli* and *C*. *neteri)*, presence of IbpB in the assemblies substantially decreased the amount of Hsp70 required for the refolding (Figs 5 and 6B). This could be beneficial for the cells at harsh or long-lasting stress conditions, when the overall cellular demand for Hsp70 is high.

Our results also suggest that the lower demand for Hsp70 in refolding of proteins from aggregates might not be the only benefit of maintaining two sHsp genes in *Eneterobacterales* genomes. All the IbpA proteins are highly specialised in binding to substrates, thus their intense production at stress conditions results in fast and efficient sequestration of aggregation-prone polypeptides in assemblies, which prevents other proteins from further co-aggregation. In *Enterobacterales*, which possess the two-sHsp system, IbpA binds to the substrates more strongly and rapidly. Thus, it provides even faster and more effective suppression of aggregation than the single-sHsp systems. At the same time, IbpB allows for easier dissociation of the tightly-bound IbpA from the assemblies. Thus, the *ibpA* duplication event at the base of *Enterobacterales* allowed the emergence of a single operon-encoded sHsps system, which combines both the efficient holdase activity and the dissociation effectiveness during stress. These are the characteristics of an ideal small heat shock protein.

Another possible gain from retaining a two-sHsp system is suggested by our results with MDH as a substrate. IbpA alone did not allow formation of small-size assemblies with MDH. Simultaneous presence of IbpB with IbpA was necessary for efficient formation of the assemblies, even though IbpB itself has a low binding potential (S3 Fig). This suggests, that for a certain pool of substrates, both IbpA and IbpB might be required to beneficially moderate the aggregation process. In consequence, the two-sHsp system’s spectrum of substrates could be broaden.

It is tempting to speculate that during the evolution of γ-proteobacteria the sequestration of polypeptides during stress (holdase function of sHsps) was fundamental. This is supported by the fact that all distantly related IbpAs bind to the substrates *in vitro*, but do not promote substrates recovery (Figs 2, 3, 5 and 6). Consequently, *in vivo* all analysed IbpAs, but not IbpB, restored the growth of bacteria under limited DnaK availability (Fig 4), suggesting higher importance of holdase function over refolding promotion.

In the ancestor of *Enterobacterales* though, fast and efficient refolding of proteins from aggregates also became desired. Thus, after duplication, IbpB acquired new properties allowing easier dissociation of sHsps from the aggregates to initiate efficient substrate refolding. Consistently, all the post-duplication bacteria contain sHsps with enhanced dissociation properties. Interestingly, in *Erwiniaceae*, IbpA exhibited an IbpB-like behaviour: it showed the lowest ability to form assemblies as compared to other IbpA proteins and the most effective dissociation from the assemblies among the analysed IbpA proteins (Figs 2 and 3). Both the holdase activity and effective dissociation in a single IbpA_*Ea*_ resulted in efficient refolding of the luciferase from the assemblies at the limited Hsp70 concentration (Fig 5). Presumably, such properties of IbpA from an ancestor of *Erwiniaceae* allowed the *ibpB* gene to be lost.

The cooperation of more than one sHsp in cellular defence against protein misfolding stress is not restricted to *Enterobacterales*. In plants, there are many classes of sHsps and several classes have multiple members, which can form heterooligomers [44, 45]. These sHsps heterooligomers interact with aggregating substrates. Similarly in humans, sHsps that have been reported to display chaperone activity, have also been shown to interact with at least one other sHsp [46]. A model example of such behaviour are αA- and αB-crystallins in human, the products of *CRYAA* and *CRYAB* genes, which co-assemble to form α-crystallin in eye lens [47]. It seems that αA-crystallin is the result of gene duplication with a specialized function in eye lens while the product of the other gene copy, αB-crystallin is also widely expressed in other tissues. Thus, the cooperation of two sHsps is common in different organisms and can be considered as an example of convergent evolution. On the other hand, there are multiple examples showing that two sHsps are working in parallel independently of each other, as reported for Hsp20.2 and Hsp17.7 from *Deinococcus radiodurans* [48] or Hsp26 and Hsp42 from *Saccharomyces cerevisiae* [49]. Recently, it has been reported that most sHsp paralogs related to an oligomeric ancestor have evolved not to form complexes with each other, and the lack of heterooligomerization correlates with acquisition of distinct functions [50]. IbpA and IbpB are then a contrasting example of proteins which retained their ability to interact with each other after a gene duplication event (23,41). Moreover, the functional divergence of individual sHsps has led to an improved performance of the system.

## Materials and methods

### Phylogenetic reconstruction

50 γ-proteobacteria proteomes from OMA database [51] were selected with maximum divergence. Using OMA we were able to identify 20,982 Orthology Groups (= OGs; sets of genes in which all representatives are Orthologous to all other members). From these we selected OGs with a minimum of 25 species represented (>50% of species with a member of the OG). To improve the overall quality of the concatenated alignment the 2 least complete or redundant taxa were now deleted (had over 40% of missing data). 1,489 OGs were kept at this stage, aligned using Clustal Omega v1.2.2 and concatenated into 200,800 amino acids alignment [52]. The positions with more than 10% of missing data were removed, which resulted in 163,081 amino acids alignment. The γ-proteobacteria species phylogeny was reconstructed using the maximum likelihood (ML) approach using RAxML 8.2.10 [53] with general time reversible model of amino acid substitution and GAMMA model of rate heterogeneity (CAT + GTR) with 1,000 ML searches and with 1000 rapid bootstrap replicates. sHsp genes were localized in the bacterial genomes using reciprocal-best-BLAST algorithm using both IbpA and IbpB as a query.

### sHsps phylogeny

The protein sequences of the IbpA and IbpB orthologs were obtained from OMA Hierarchical Orthologous Groups [54]. The sequences were aligned using Clustal Omega v1.2.2 with default parameters [52]. The alignment was corrected and trimmed manually. To infer protein phylogeny, 10,000 ML searches were performed using RAxML v8.2.10 [53] with 100 rapid bootstrap replicates with constrained for species containing both IbpA and IbpB to prevent Long Branch Attraction artifact. LG model of amino acid substitution and GAMMA model of rate heterogeneity with four discrete rate categories and the estimate of proportion of invariable sites (LG + I + G) [55] was determined as the best-fit model by ProtTest v3.2 following Akaike criterion [56] was selected for the analysis.

### Proteins

Purifications of DnaK, DnaJ, GrpE, ClpB, IbpA, His-IbpB proteins were performed as described previously [23]. The purification of C-His-tagged luciferase (used only in BLI experiments) was based on the interaction of the His-tag with Ni-NTA agarose.

IbpA from *V*. *harveyi* was overproduced in *E*. *coli* BL21(DE3) from pET15b plasmid. Cells were then lysed using French press in buffer A (50 mM Tris-HCl pH 7.5, 50 mM NaCl, 10% Glycerol, 0.5 mM EDTA, 5 mM βMe) and centrifuged (75 000 g, 25 min, 4°C). The soluble fraction was applied onto Q-Sepharose resin and flow-through fractions containing IbpA_*Vh*_ were dialysed against buffer B (50 mM Tris-HCl pH 7.5, 50 mM NaCl, 10% Glycerol, 5 mM βMe, 6 M Urea) prior to a next round of Q-Sepharose chromatography in denaturing conditions (elution in buffer B with 50 to 500 mM NaCl gradient). IbpA_*Vh*_ was then dialysed against buffer B and resubjected to Q-Sepharose chromatography, but this time eluted with a pH gradient (gradient from buffer B with Tris-HCl pH 7.5 to buffer B with citrate buffer pH 5.0 instead of Tris-HCl). Finally Urea was slowly dialysed out against buffer C (50 mM Tris-HCl pH 8.5, 150 mM KCl, 10% Glycerol, 5 mM βMe). IbpA from *E*. *amylovora was* purified using the same protocol.

*C*. *neteri ibpA* and *ibpB* together with *A*. *hydrophila ibpA* genes were ordered from Genscript (cloned into pET3a plasmids). *C*. *neteri* IbpA and *A*. *hydrophila* IbpA were purified as follows: sHsps were overproduced in *E*. *coli* BL21(DE3) from pET3a plasmids. Cells were then lysed using French press in buffer A and centrifuged (75 000 g, 25 min, 4°C). sHsps were extracted from insoluble fraction by shaking in buffer B, sample was then centrifuged (75 000 g, 25 min, 4°C). sHsps-containing supernatant was applied onto Q-Sepharose in denaturing conditions and eluted in buffer B with 50 to 500 mM NaCl gradient. Fractions containing sHsps were then slowly dialysed against buffer C.

*C*. *neteri* IbpB was overproduced in *E*. *coli* BL21(DE3) from pET3a plasmid. Cells were then lysed using French press in buffer A and centrifuged (75 000 g, 25 min, 4°C). Proteins were then salted-out with 50%-saturated ammonium sulphate, centrifuged (75 000 g, 45 min, 4°C) and dissolved in buffer B prior dialysis to buffer B. Sample was then applied in denaturing conditions onto Q-Sepharose and eluted in buffer B with 50 to 500 mM NaCl gradient. Fractions containing IbpB were then dialysed to buffer C1 (50 mM Tris-HCl pH 8.5, 50 mM KCl, 10% Glycerol, 5 mM βMe) and negatively purified using Q-Sepharose resin. Finally, the sample was dialysed against buffer C.

### DLS measurements

Particle size was determined using Malvern Instruments ZetaSizer Nano S dynamic light scattering instrument. Measurements were taken in buffer D (50 mM Tris-HCl pH 7.5, 150 mM KCl, 20 mM Mg acetate, 2 mM DTT). Luciferase was present at a fixed 1.5 μM concentration and sHsp concentrations ranged from 0 to 10 μM. The conditions were as follows: measurement volume—40 μl, scattering angle—173°, wavelength—633 nm, temperature—25°C. For every data point minimum 10 measurements of ten 10s runs were averaged and particle size distribution was calculated by fitting to 70 size bins between 0.4 and 10,000 nm. Results are shown as average diameter of the main peak with SD (at least 80% of total measured particle mass was contained in main peak) plotted against sHsp concentration. IbpA_*Ec/Cn*_ and IbpB_*Ec/Cn*_ (IbpAB_*Ec/Cn*_) were always used in fixed 1:2 ratio when together, concentration depicted is for both proteins in total.

### Sedimentation analysis of assemblies formation

Luciferase (1 μM) was denatured at 44°C for 10 min in buffer D in the presence of 5 μM sHsps (1.66 μM IbpA_*Ec*_ and 3,34 μM IbpB_*Ec*_ in case of IbpAB_*Ec*_). To verify sHsps ability to form assemblies, 150 μl of each sample was applied on a 3.6 ml 10–60% glycerol gradient in buffer E (50 mM Tris-HCl pH 7.5, 150 mM NaCl, 20 mM Mg acetate, 2 mM DTT). Samples were then centrifuged at 10°C in a Beckman SW 60 rotor at 160,000 g for 1 h, fractions were collected from the top. Protein distribution in each fraction was verified by SDS–PAGE followed by Oriole (Bio-Rad) fluorescent staining.

### BLI experiments

BLI experiments were performed on BLItz device using Dip and Read Ni-NTA (NTA) Biosensors (ForteBio) at room temperature with 2000 rpm mixing. Basal anchoring luciferase layer was immobilized on the sensor in denaturing conditions with 0.6 mg/ml C-His-tagged luciferase in buffer F (6M Urea, 50 mM Tris pH 7.5, 150 mM KCl, 20 mM MgCl2, 2 mM DTT) for 15 min. Excess luciferase and denaturant were washed away with buffer D. The top luciferase aggregate layer was formed by transferring the sensor with the anchoring layer to a test tube with 0.1 mg/ml C-His-tagged luciferase in buffer D and subsequent incubation in 44°C for 10 min. Next, the sensor was transferred back to the BLItz device where non-bound luciferase aggregates were washed away in buffer D for 10 min. 5 μM sHsps in buffer D (or 1.66 μM IbpA_*Ec*_ with 3,34 μM IbpB_*Ec*_ in case of IbpAB_*Ec*_) were heat-activated at 44°C for 5 min and immediately transferred to the BLItz instrument. sHsps were allowed to bind the immobilized luciferase aggregate for 10 min then dissociated either in buffer D or in the presence of Hsp70 chaperone system (limiting and saturating concentration) for 1 h. Proteins bound to the sensor were stripped with Laemmli buffer before and after Hsp70 system incubation (separate experiment) and subjected to SDS-PAGE followed by Western blot or Oriole staining.

### Drop test experiments

*E*. *coli* MC4100 P_IPTG_ *dnaKJ* Δ*ibpAB* strains [4] carrying pBR322 plasmid with cloned *E*. *coli ibpAB* operon or with stop codon introduced in either *ibpA* or *ibpB* gene (F4Amber in both cases) or with *ibpA* gene seamlessly replaced with *E*. *amylovora* or *V*. *harveyi ibpA* (additionally with stop codon in *ibpB* as above) were grown at 30°C in LB supplemented with 1mM IPTG until OD≈1. Serial dilutions were plated on LA medium supplemented with 100 μM, 75 μM, 50 μM or without IPTG and grown for 24 h in 37°C.

### Luciferase denaturation and refolding experiments

Luciferase (1.5 μM) was denatured at 44°C for 10 min in buffer D in the presence or absence of 10 μM sHsps as indicated (3 μM IbpA_*Ec/Cn*_ and 7 μM IbpB_*Ec/Cn*_ in case of IbpAB_*Ec/Cn*_). Protein refolding was started by 40-fold dilution of denatured luciferase in the Hsp70-Hsp100 chaperone cocktail. Unless noted otherwise, the chaperone concentrations used were as follows: Limiting Hsp70—DnaK 0.7 μM, DnaJ 0.28 μM, GrpE 0.21 μM, ClpB 2 μM; saturating Hsp70—DnaK 3.5 μM, DnaJ 1.4 μM, GrpE 1.05 μM, ClpB 2 μM. All assays were performed in the presence of an ATP-regenerating system (18 mM creatine phosphate, 0.1 mg/ml creatine kinase, 5 mM ATP). The disaggregation reaction was carried out at 25°C. Luciferase activity was measured at time points using a Sirius Luminometer (Berthold), normalized and presented as a mean ±SD from at least 3 independent experiments. 100% activity is defined as the maximal activity obtained during the refolding of luciferase-IbpAB assemblies. The normalisation procedure was performed in this way since the native luciferase (Promega) contains the fraction of inactive protein and the activity of refolded luciferase often exceeded the activity of native luciferase preparation used for denaturation (by approximately 20–30%).

### Hsp70-dependent release of sHsps from assemblies

Luciferase (3 μM) was denatured at 44°C for 10 min in buffer D (50 mM Tris, pH 7.5, 150 mM KCl, 20 mM Mg acetate, 2 mM DTT) in the presence of IbpA_*Ec*_ (6 μM) with and without His-IbpB_*Ec*_ (14 μM). Alternatively, 1.5 μM Luciferase was denatured at 44°C for 10 min in buffer D in presence of: 3 μM IbpA_*Cn*_ with or without 7 μM IbpB_*Cn*_ or 10 μM IbpA_*Vh*_ or 10 μM IbpA_*Ah*_

To isolate the IbpA/IbpAB–luciferase assemblies from unbound sHsps, 400 μl of preformed assemblies was applied on a 3.4 ml 10–60% glycerol gradient in buffer D. Samples were centrifuged at 10°C in a Beckman SW 60 rotor at 160,000 g for 1 h, fractions were collected from the top. Protein distribution in each fraction was verified by SDS–PAGE followed by Oriole (Bio-Rad) fluorescent staining. Fractions containing IbpAB- and IbpA-luciferase assemblies were pooled together and the substrate concentration was determined by optical densitometry following SDS–PAGE at 21 ng/μl for Luc-IbpAB_*Ec*_, 6 ng/μl for Luc-IbpA_*Ec*_, 29 ng/μl for Luc-IbpAB_*Cn*_, 33 ng/μl for Luc-IbpA_*Cn*_, 42 ng/μl for Luc-IbpA_*Vh*_ and 42 ng/μl for Luc-IbpA_*Ah*_ relative to the luciferase concentration. To analyse the effect of chaperones on isolated assemblies, the assemblies were diluted to 1.5 ng/μl of luciferase in limiting and saturating Hsp70 system (DnaK 0.7 μM, DnaJ 0.28 μM, GrpE 0.21 μM or DnaK 3.5 μM, DnaJ 1.4 μM, GrpE 1.05 μM respectively), incubated for 45 min at room temp. and subjected to a second round of sedimentation under the conditions listed above. Fractions containing unbound sHsps (fractions 1–2; 1 for IbpA_*Ah*_) and sHsps-luciferase assemblies (fractions 4–7 for IbpA_*Ec*_ and IbpAB_*Ec*_; 3–5 for IbpA_*Cn*_ and IbpA_*Vh*_; 4–6 for IbpAB_*Cn*_ and 2–4 for IbpA_*Ah*_) were pooled and precipitated with trichloroacetic acid. Pellets were then resuspended in Laemmli buffer and together with bottom fraction analysed by Western blot using antibodies against IbpA_*Ec*_ (for IbpA_*Ec*_; IbpA_*Cn*_ and IbpA_*Ah*_) and against IbpA_*Vh*_.

### Isolation of aggregated protein fraction

*E*. *coli* MC4100 Δ*ibpA* and Δ*ibpB* strains were developed by introducing single stop codons on the chromosomal DNA at positions M12 (*ibpB*) and R11 (*ibpA*) respectively using no-SCAR method [57]. *E*. *coli* MC4100 Δ*ibpAB* strain [4] was kindly provided by B. Bukau.

*E*. *coli* strains were cultured at 30°C in Luria Broth (LB). Temperature-shift experiments were performed in shaking water baths. The cells were grown overnight, diluted 1:50 in fresh LB and grown until OD = 1. Next, cultures were pre-shocked at 42°C for 10 min, then heat-shocked at 48°C for 5 min. Finally cells were shifted to 30°C for recovery. Aliquots of bacterial cultures (10 ml) were rapidly cooled to 0°C in ice-water bath and centrifuged for 6 min at 4000g to harvest the cells. Pellets were resuspended in 80 μl of buffer G (10 mM KPi pH 6.5; 1 mM EDTA; 20% Sucrose; 1 mg/ml Lysozyme) and incubated on ice for 30 min. Cells were then lysed by (Qsonica Q700, tip no. 4418, amplitude 4). Cell debris was removed by 5 min centrifugation at 1000 g at 4°C. Supernatant was supplemented with IGEPAL CA-630 non-ionic detergent to final concentration of 4% (v/v) and vortexed vigorously. Aggregated protein fraction was harvested by centrifugation at 20000 g at 4°C for 20 min. Pellet fractions were washed twice with buffer I (10 mM KPi pH 6.5; 1 mM EDTA; 5% (v/v) IGEPAL CA-630) in order to remove membrane fraction and finally washed with buffer H for detergent removal. The resulting pellet was resuspended in 40 μl of Laemmli buffer prior to SDS-PAGE and WB analysis.

### SDS-PAGE and Western-blot analysis

Gel electrophoresis was performed using self-prepared 15% polyacrylamide gels or 4–20% ExpressPlus PAGE Gels (Genescript) according to standard procedures and stained with Coomassie brilliant blue or Oriole (Bio-Rad) fluorescent stain. Gel electrophoresis for separation of IbpA_*Ec*_ from IbpB_*Ec*_ was performed using 15% polyacrylamide gels supplemented with 6M Urea. Immunoblotting was performed according to the standard procedures, using rabbit anti-sera specific for IbpAs and IbpAB as primary antibody, and developed with SuperSignal West Pico Chemiluminescent Substrate (Thermo Scientific), using (H+L) HRP conjugated anti-rabbit IgG (Bio-Rad) as secondary antibodies. Developed immunoblots were scanned using ChemiDoc MP Imaging System (Bio-Rad) and quantified with ImageLab (Bio-Rad) software.

## Supporting information

S1 Fig Phylogeny of 48 Gammaproteobacteria species based on 1,489 genes. Analysis of 163,081 unambiguously aligned positions with 10% missing data. Tree was reconstructed using Γ+GTR model under a Maximum Likelihood analysis. Monophyly of *Enterobacterales* is supported. *Erwiniacea* is a sister group to *Enterobacteriaceae*. *Vibionaceae* and *Enterobacterales* are sister groups. Nodes with BP = 100 are marked with a star. Scale bar, substitutions per position.
(TIF)

S2 Fig sHsps phylogeny in Gammaproteobacteria. Full Maximum Likelihood tree of 93 sHsps amino acid sequences from Gammaproteobacteria, calculated with LG + I + G model in RAxML based on 82 amino acid positions. Scale is in expected amino acid substitutions per site. Level of bootstrap support is indicated with dots, bootstrap > 90 in magenta, bootstrap ≤ 90 and > 70 in yellow, bootstrap ≤ 70 in green. IbpA sequences are in red and IbpB sequences are in blue. Proteins used in experiments are marked with brackets.
(TIF)

S3 Fig IbpA*_Ec_* and IbpB*_Ec_* ability to protect luciferase, malate dehydrogenase (MDH) and citrate synthase (CS) from aggregation. Luciferase (1.5 μM), malate dehydrogenase (2 μM) or citrate synthase (2 μM) were mixed with IbpA_*Ec*_ (3 μM, red), IbpB_*Ec*_ (7 μM, blue), both IbpA_*Ec*_ and IbpB_*Ec*_ (3 μM and 7 μM respectively, green) or none sHsps (black) in room temperature (0°C in case of luciferase) and injected to preheated (temp. as indicated) spectrofluorometric cuvettes prior to scattering measurement. Used wavelengths were 605 nm for luciferase and citrate synthase and 565 nm for malate dehydrogenase.
(TIF)

S4 Fig DnaK expression in *E. coli* MC4100 PI_PTG_ *dnaKJ* Δ*ibpAB* strain. *E*. *coli* cells were grown in LB supplemented with chloramphenicol at 30°C overnight. Cultures were then diluted in fresh LB with chloramphenicol and indicated concentration of IPTG and grown in 37°C for 3 h prior harvesting. Cells were then subjected to SDS-PAGE and stained with Coomassie Brilliant Blue.
(TIF)

S5 Fig sHsps levels in drop test experiment. *E*. *coli* MC4100 P_IPTG_ *dnaKJ* Δ*ibpAB* strains carrying pBR322 plasmid with indicated genes under *ibpAB* heat shock promoter were grown in LB medium supplemented with ampicillin and 100 μM IPTG at 37°C until late logarithmic phase. Then cells were harvested and subjected to SDS-PAGE and Western blot analysis. Plasmids were constructed in a way that they carried entire *E*.*coli ibpAB* operon with indicated *ibpA* genes seamlessly introduced instead of *E*. *coli ibpA* (or unmodified) accompanied with *ibpB* F4Amber. For E. coli *ibpB*-only plasmid construction stop codon was introduced in *ibpA* gene at position F4. The IbpA_*Ec*_ level was assessed by Western blot. The level of other sHsps was assessed on Commassie blue stained SDS-PAGE using respected purified proteins as markers.
(TIF)

S6 Fig Isolation of sHsps-luciferase assemblies by sedimentation. Luciferase (3 μM) and IbpA_*Ec*_ (6 μM) or IbpAB_*Ec*_ (6 μM and14 μM, respectively) were aggregated at 48°C for 10 min and subjected for glycerol gradient sedimentation (Beckman SW60Ti, 40 000 rpm, 1h, 10°C). Fractions were collected from the top and analyzed by SDS-PAGE followed by Oriole staining. Fractions containing luciferase-IbpA_*Ec*_ and -IbpAB_*Ec*_ assemblies were pooled and stored in -70°C for further use.
(TIF)

S7 Fig IbpB presence in CS-IbpAB assemblies allows for efficient Hsp70-dependent dissociation of IbpA from assemblies. Citrate Synthase (1.5 μM) and IbpA_*Ec*_ (3 μM) or IbpAB_*Ec*_ (3 μM and 7 μM, respectively) were aggregated at 52°C for 10 min and subjected for glycerol gradient sedimentation (Beckman SW60Ti, 40 000 rpm, 1 h, 10°C) for isolation from excess unbound sHsps and aggregates. Isolated CS-sHsps assemblies were incubated with buffer or limiting (DnaK 0.7 μM; DnaJ 0.28 μM; GrpE 0.21 μM) or saturating (DnaK 3.5 μM; DnaJ 1.4 μM; GrpE 1.05 μM) Hsp70 machinery concentration followed by glycerol gradient sedimentation. Fractions were collected from the top, pooled (top—fractions containing free sHsps; middle—fractions containing sHsps-luciferase assemblies; bottom–material recovered from the bottom of centrifugation tube) and analyzed by Western blot with IbpA antibodies following SDS-PAGE.
(TIF)
