# Supplementary figures and images for: Duplicate divergence of two bacterial small heat shock proteins reduces the demand for Hsp70 in refolding of substrates
Source: PLoS Genet. 2019 Oct 25;15(10):e1008479. doi: 10.1371/journal.pgen.1008479 (PMC6834283; doi:10.1371/journal.pgen.1008479)

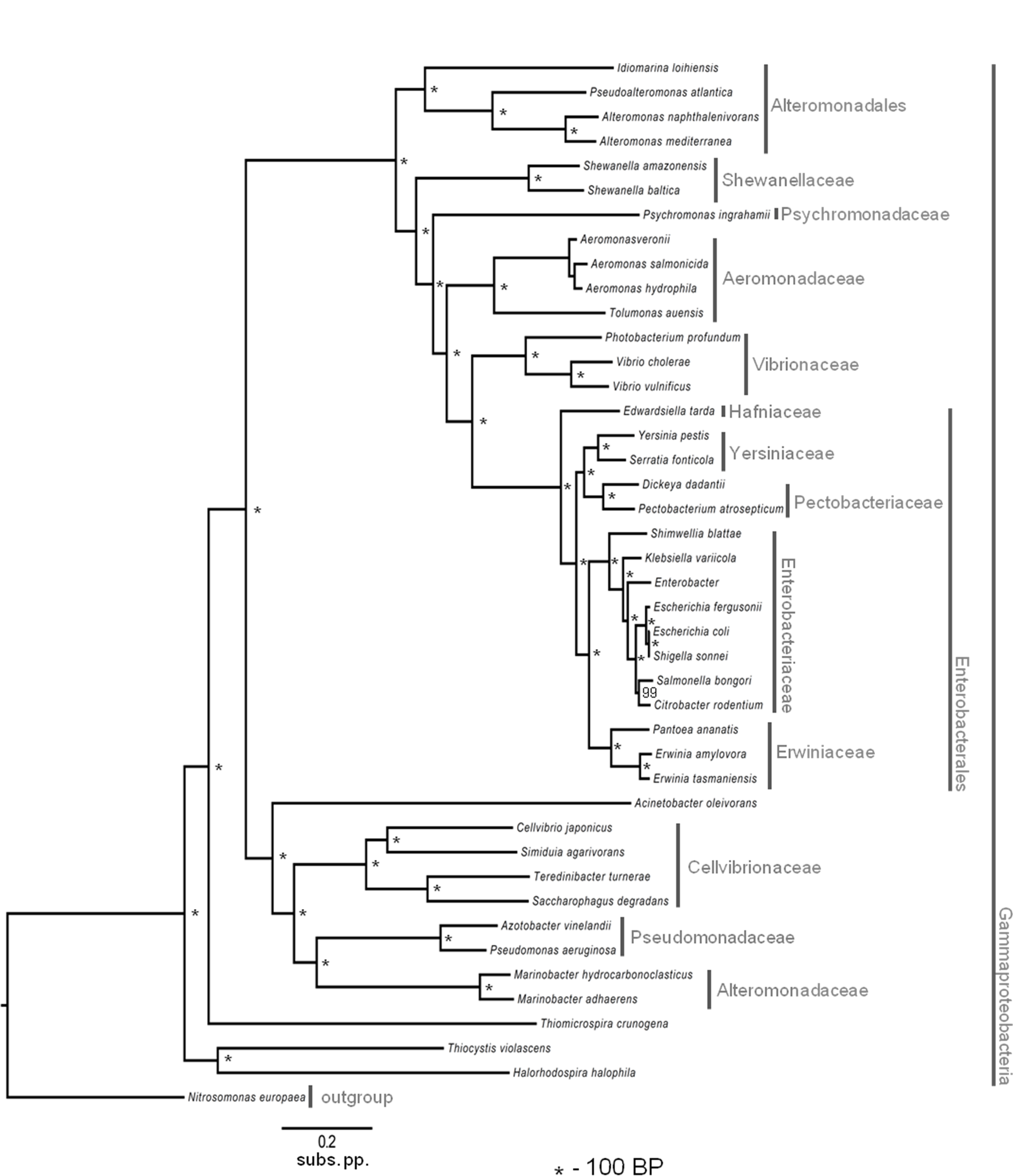

Supplement: S1 Fig — Analysis of 163,081 unambiguously aligned positions with 10% missing data. Tree was reconstructed using Γ+GTR model under a Maximum Likelihood analysis. Monophyly of Enterobacterales is supported. Erwiniacea is a sister group to Enterobacteriaceae. Vibionaceae and Enterobacterales are sister groups. Nodes with BP = 100 are marked with a star. Scale bar, substitutions per position. (TIF) [file pgen.1008479.s001.tif]

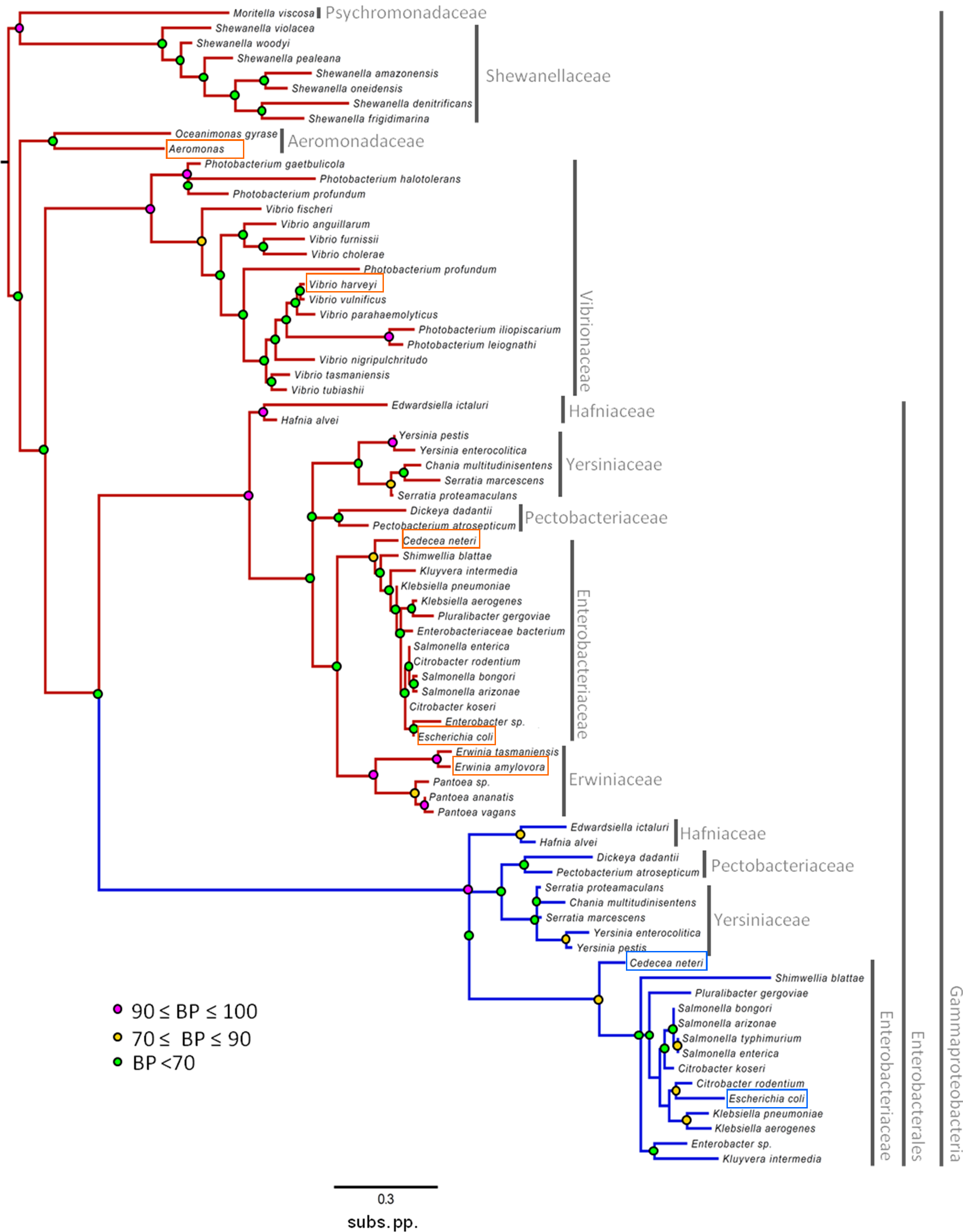

Supplement: S2 Fig — Full Maximum Likelihood tree of 93 sHsps amino acid sequences from Gammaproteobacteria, calculated with LG + I + G model in RAxML based on 82 amino acid positions. Scale is in expected amino acid substitutions per site. Level of bootstrap support is indicated with dots, bootstrap > 90 in magenta, bootstrap ≤ 90 and > 70 in yellow, bootstrap ≤ 70 in green. IbpA sequences are in red and IbpB sequences are in blue. Proteins used in experiments are marked with brackets. (TIF) [file pgen.1008479.s002.tif]

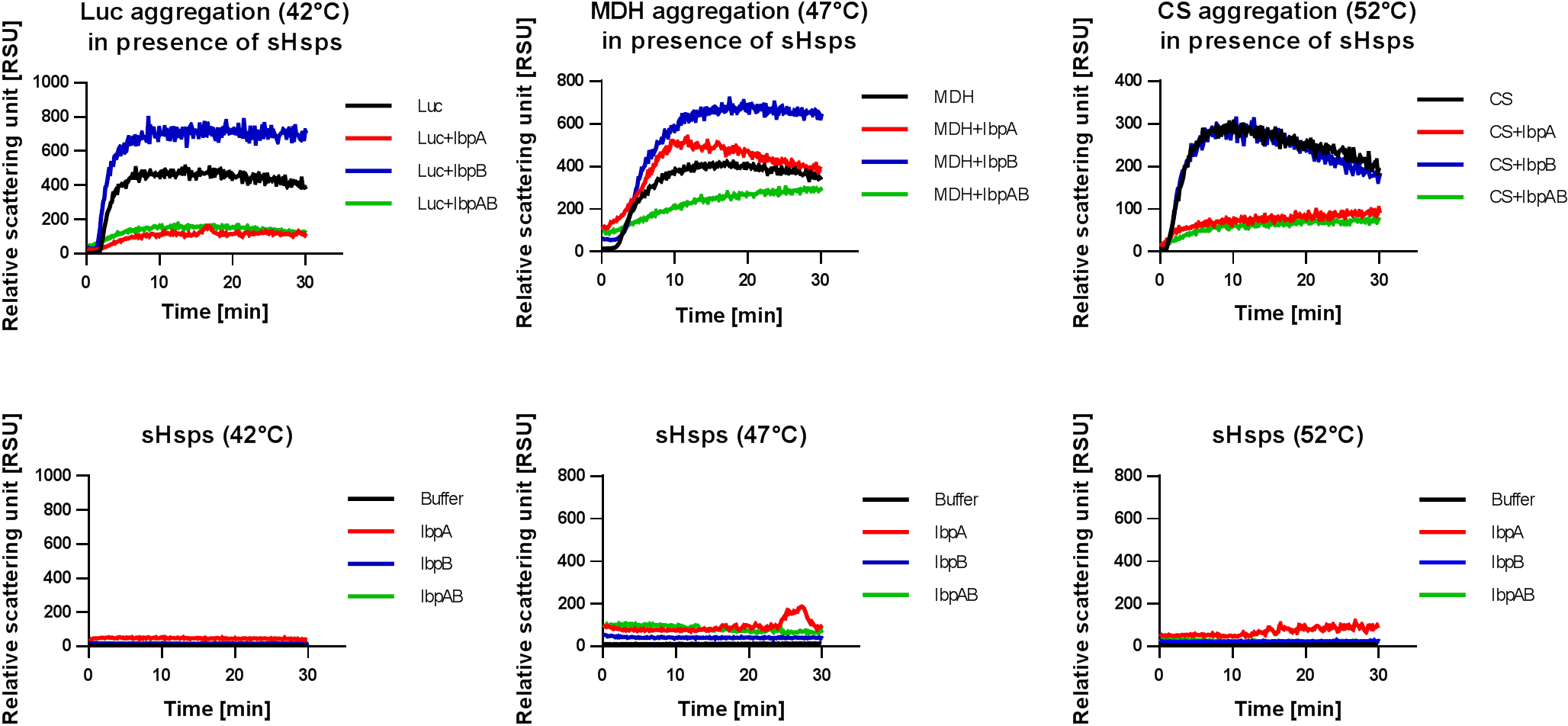

Supplement: S3 Fig — Luciferase (1.5 μM), malate dehydrogenase (2 μM) or citrate synthase (2 μM) were mixed with IbpAEc (3 μM, red), IbpBEc (7 μM, blue), both IbpAEc and IbpBEc (3 μM and 7 μM respectively, green) or none sHsps (black) in room temperature (0°C in case of luciferase) and injected to preheated (temp. as indicated) spectrofluorometric cuvettes prior to scattering measurement. Used wavelengths were 605 nm for luciferase and citrate synthase and 565 nm for malate dehydrogenase. (TIF) [file pgen.1008479.s003.tif]

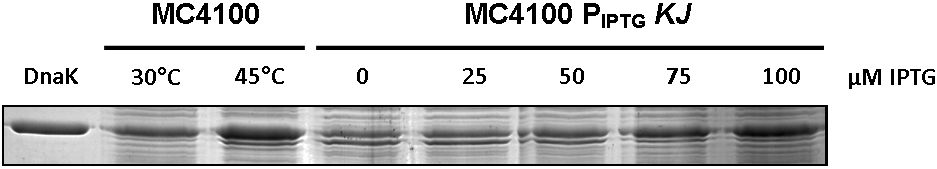

Supplement: S4 Fig — E. coli cells were grown in LB supplemented with chloramphenicol at 30°C overnight. Cultures were then diluted in fresh LB with chloramphenicol and indicated concentration of IPTG and grown in 37°C for 3 h prior harvesting. Cells were then subjected to SDS-PAGE and stained with Coomassie Brilliant Blue. (TIF) [file pgen.1008479.s004.tif]

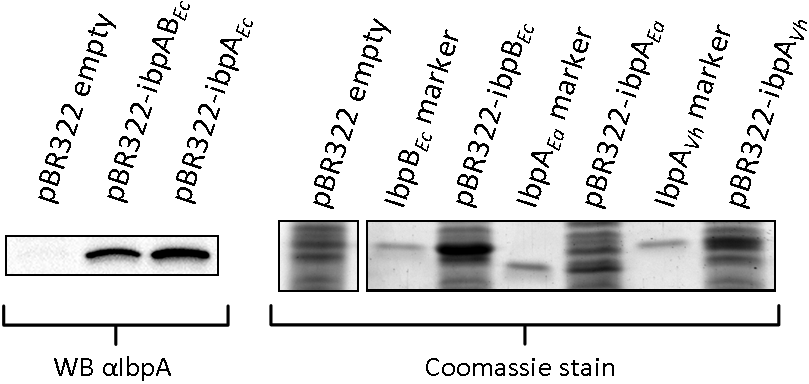

Supplement: S5 Fig — E. coli MC4100 PIPTG dnaKJ ΔibpAB strains carrying pBR322 plasmid with indicated genes under ibpAB heat shock promoter were grown in LB medium supplemented with ampicillin and 100 μM IPTG at 37°C until late logarithmic phase. Then cells were harvested and subjected to SDS-PAGE and Western blot analysis. Plasmids were constructed in a way that they carried entire E.coli ibpAB operon with indicated ibpA genes seamlessly introduced instead of E. coli ibpA (or unmodified) accompanied with ibpB F4Amber. For E. coli ibpB-only plasmid construction stop codon was introduced in ibpA gene at position F4. The IbpAEc level was assessed by Western blot. The level of other sHsps was assessed on Commassie blue stained SDS-PAGE using respected purified proteins as markers. (TIF) [file pgen.1008479.s005.tif]

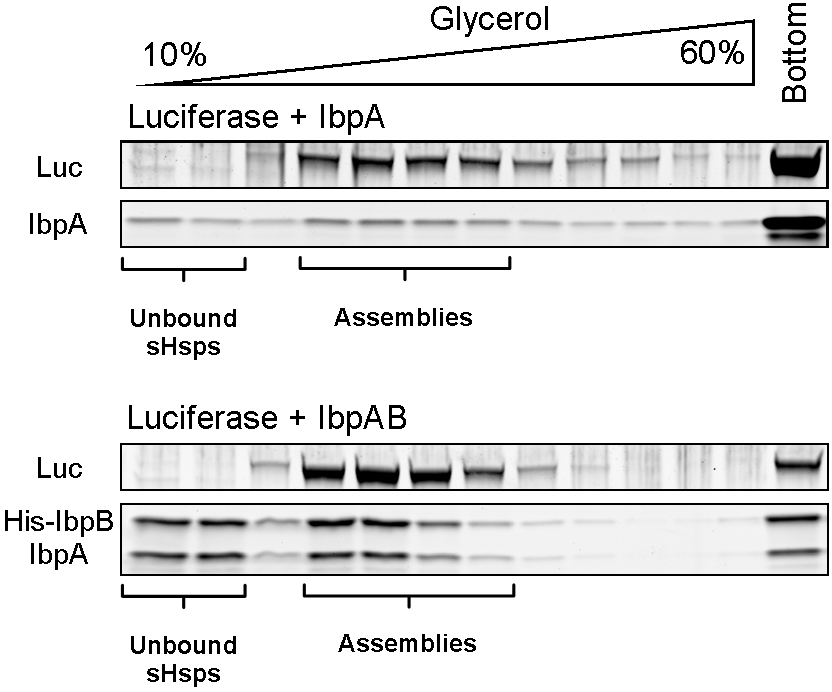

Supplement: S6 Fig — Luciferase (3 μM) and IbpAEc (6 μM) or IbpABEc (6 μM and14 μM, respectively) were aggregated at 48°C for 10 min and subjected for glycerol gradient sedimentation (Beckman SW60Ti, 40 000 rpm, 1h, 10°C). Fractions were collected from the top and analyzed by SDS-PAGE followed by Oriole staining. Fractions containing luciferase-IbpAEc and -IbpABEc assemblies were pooled and stored in -70°C for further use. (TIF) [file pgen.1008479.s006.tif]

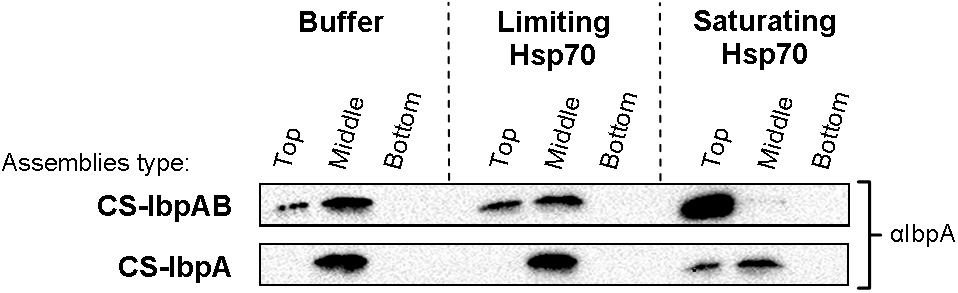

Supplement: S7 Fig — Citrate Synthase (1.5 μM) and IbpAEc (3 μM) or IbpABEc (3 μM and 7 μM, respectively) were aggregated at 52°C for 10 min and subjected for glycerol gradient sedimentation (Beckman SW60Ti, 40 000 rpm, 1 h, 10°C) for isolation from excess unbound sHsps and aggregates. Isolated CS-sHsps assemblies were incubated with buffer or limiting (DnaK 0.7 μM; DnaJ 0.28 μM; GrpE 0.21 μM) or saturating (DnaK 3.5 μM; DnaJ 1.4 μM; GrpE 1.05 μM) Hsp70 machinery concentration followed by glycerol gradient sedimentation. Fractions were collected from the top, pooled (top—fractions containing free sHsps; middle—fractions containing sHsps-luciferase assemblies; bottom–material recovered from the bottom of centrifugation tube) and analyzed by Western blot with IbpA antibodies following SDS-PAGE. (TIF) [file pgen.1008479.s007.tif]
